# Tumor Site‐Specific In Vivo Theranostics Enabled by Microenvironment‐Dependent Chemical Transformation and Self‐Amplifying Effect

**DOI:** 10.1002/advs.202409506

**Published:** 2024-11-29

**Authors:** Yunfei Zuo, Pei Li, Wen‐Jin Wang, Changhuo Xu, Shuting Xu, Herman H. Y. Sung, Jianwei Sun, Guorui Jin, Weiping Wang, Ryan T. K. Kwok, Jacky W. Y. Lam, Ben Zhong Tang

**Affiliations:** ^1^ Department of Chemistry Hong Kong Branch of Chinese National Engineering Research Center for Tissue Restoration and Reconstruction Division of Life Science State Key Laboratory of Molecular Neuroscience and Department of Chemical and Biological Engineering The Hong Kong University of Science & Technology Clear Water Bay Kowloon Hong Kong 999077 P. R. China; ^2^ National Clinical Research Center for Infectious Diseases Shenzhen Third People's Hospital Southern University of Science and Technology Shenzhen Guangdong 518112 China; ^3^ China Clinical Translational Research Center of Aggregation‐Induced Emission The Second Affiliated Hospital School of Medicine School of Science and Engineering Shenzhen Institute of Aggregate Science and Technology The Chinese University of Hong Kong Shenzhen (CUHK‐Shenzhen) Guangdong 518172 China; ^4^ MOE Frontiers Science Center for Precision Oncology Faculty of Health Sciences University of Macau Macao 999078 China; ^5^ Department of Pharmacology and Pharmacy Li Ka Shing Faculty of Medicine The University of Hong Kong Hong Kong 999077 China; ^6^ The Key Laboratory of Biomedical Information Engineering of Ministry of Education School of Life Science and Technology Xi'an Jiaotong University Xi'an 710049 China

**Keywords:** aggregation‐induced emission, autocatalytic reaction, cancer theranostics, hydroxyl radical probes, specific targeting

## Abstract

Precise tumor diagnosis and treatment remain complex challenges. While numerous fluorescent probes have been developed for tumor‐specific imaging and therapy, few exhibit effective function in vivo. Herein, a probe called TQ‐H_2_ is designed that can realize robust theranostic effects both in vitro and in vivo. In vitro, TQ‐H_2_ specifically targets the lysosome and reacts with hydroxyl radical (·OH) to generate TQ‐HA, which lights up the cells. TQ‐HA generates reactive oxygen species (ROS) under light irradiation, enabling the simultaneous induction and monitoring of apoptosis and ferroptosis in tumor cells. Remarkably, TQ‐HA also acts as a self‐amplifier, autocatalytically activating TQ‐H_2_ by generating ·OH under light exposure. This self‐amplification aligns with the tumor microenvironment, where TQ‐H_2_ undergoes chemical transformation, distinguishing tumors from healthy tissue via near‐infrared (NIR) fluorescence imaging. Furthermore, ROS generated by TQ‐HA effectively kills tumor cells and inhibits tumor growth without harming normal cells. This study offers a promising strategy for targeted tumor theranostics using self‐amplifying microenvironment‐responsive fluorescent probes.

## Graphical abstract

TQ‐H_2_ is a highly specific, ultrasensitive, and fast‐responding probe for hydroxyl radicals (·OH), producing TQ‐HA, which emits deep red fluorescence and activates in cells. Under light irradiation, TQ‐HA further amplifies TQ‐H_2_ activation by generating additional ·OH, creating a cyclic amplification. This autocatalytic reaction enhances the contrast between tumor and normal tissues, offering a promising platform for tumor‐specific in vivo phototherapy and theranostics.

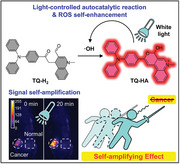

## 1 Introduction

Cancer is a leading cause of death worldwide and causes millions of new cases and deaths each year.^[^ ^1^, ^2^ ^]^ Traditional treatments, including surgery, radiation, and chemotherapy, face limitations.^[^ ^3^, ^4^ ^]^ For instance, surgical tumor resection requires precise imaging, but distinguishing tumors from healthy tissue often relies on the surgeon's naked eye, risking incomplete removal and recurrence.^[^ ^5^, ^6^, ^7^ ^]^ Similarly, chemotherapy lacks tumor specificity, with over 99% of drugs absorbed by healthy organs, leading to severe side effects, inadequate concentrations in deep tumor tissue, and increased drug resistance.^[^ ^8^, ^9^, ^10^ ^]^ These challenges highlight the need for precise theranostic with the ability to differentiate tumors and augment their effective dose at the tumor site with high toxicity.^[^ ^11^ ^]^

Photosensitizers (PSs)‐based tumor therapies not only enable diagnostic imaging by fluorescence, photoacoustic, or photothermal modalities, but also facilitate non‐invasive tumor ablation through ROS‐based photodynamic therapy (PDT) or heat‐based photothermal therapy (PTT). However, most PSs operate in a fluorescence “on” mode, resulting in a low signal‐to‐noise ratio (SNR) and significant background interference in biological imaging, making tumor distinguish challenging.^[^ ^12^, ^13^, ^14^ ^]^ Additionally, many PSs lack tumor specificity, accumulating in metabolic organs like the liver and kidneys, leading to side effects and requiring patients to avoid sunlight and even indoor light post‐treatment.^[^ ^4^, ^15^ ^]^

To improve specificity, PSs can be conjugated to antibodies or ligands targeting tumor cells.^[^ ^3^ ^]^ However, this approach is limited to certain cancer types and presents challenges such as potential allergic reactions, high production costs, and complex logistics for large‐scale use. Therefore, activatable PSs that respond to specific biomarkers in the tumor microenvironment have gained widespread attention for achieving high SNR diagnostics and efficient tumor ablation.^[^ ^4^, ^5^, ^6^, ^14^, ^15^, ^16^, ^17^ ^]^ While many probes have been developed for in vitro cancer cell discrimination,^[^ ^18^, ^19^, ^20^, ^21^, ^22^ ^]^ those with specific targeting and theranostic properties in vivo remain rare. In the complex physiological environment, distinguishing non‐targeted tissues from tumors is difficult when the concentration of biomarkers in the microenvironment is too low or if there is insufficient difference between tumor and normal tissues.^[^ ^23^, ^24^, ^25^ ^]^ Developing more sensitive activatable PSs could address this challenge, but achieving high specificity and avoiding false signals in vivo remains difficult. Signal amplification, which enhances recognition and magnifies weak input signals, is a fundamental concept in biology and engineering. Inspired by this, designing activatable PSs that respond and amplify tumor‐specific signals is an attractive strategy for cancer theranostics.

The tumor microenvironment, characterized by low pH and overproduced reactive oxygen species (ROS),^[^ ^26^, ^27^, ^28^, ^29^, ^30^, ^31^, ^32^, ^33^, ^34^ ^]^ plays a crucial role in tumor development. Hydroxyl radicals (·OH), the most reactive and oxidative ROS, are key players in tumor initiation, progression, metastasis, and cell death.^[^ ^35^, ^36^, ^37^, ^38^, ^39^, ^40^ ^]^ We noted that some dihydroquinolines have been documented in literature showing reactivity to ·OH.^[^ ^41^, ^42^, ^43^ ^]^ The authors believed that the keto group of dihydroquinolines reacts preferentially than the aldehyde group with ·OH to give oxidized products with enol functionality. On the other side, it is well‐known that molecules containing hydroxyl and aldehyde groups, particularly those with 5‐ and 6‐membered rings, undergo readily intramolecular hemiacetal formation, which is a ubiquitous phenomenon observed in biological systems.^[^ ^44^, ^45^ ^]^ For instance, glucose primarily exists as a cyclic hemiacetal under physiological conditions.^[^ ^46^ ^]^ Thus, further investigation into the molecular structures of oxidized dihydroquinoline products is warranted.

Traditional fluorescent probes often suffer from aggregation‐caused quenching (ACQ), where strong emission in dilute solutions weakens as probes aggregate.^[^ ^47^, ^48^ ^]^ In contrast, aggregation‐induced emission (AIE) luminogens (AIEgens) exhibit enhanced fluorescence and ROS generation upon aggregation or restricted intramolecular motion, showing superior PDT effects.^[^ ^4^, ^38^, ^49^, ^50^, ^51^, ^52^, ^53^, ^54^ ^]^ This makes designing probes that transforms ACQ to AIE or activate in response to the tumor microenvironment highly desirable. The fluorescence of dihydroquinolines is induced or greatly enhanced and red‐shifted upon reaction with ·OH. Depending on the substituent attached to the keto group, strong infra‐red emission can be realized in the oxidized products. Given the abundance of ·OH in the tumor tissue, dihydroquinolines offer promising scaffolds for tumor‐specific fluorescence turn‐on or ratiometric probes.

**Figure 1:**
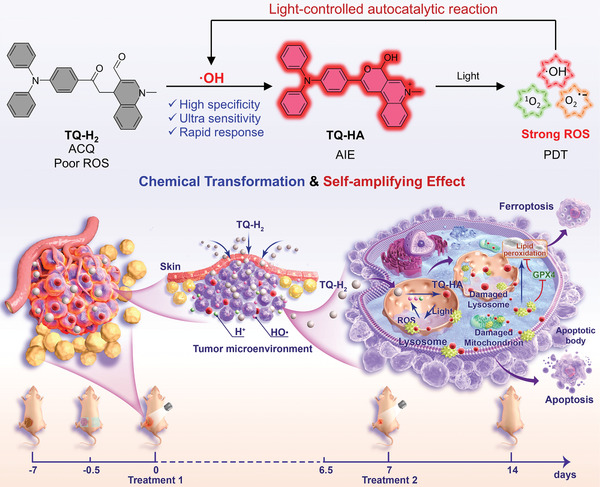
Schematic illustration of a theranostic platform for tumor site‐specific in vivo phototherapy by chemical transformation of TQ‐H_2_ to TQ‐HA and self‐amplifying effect of TQ‐HA with photo‐control.

Here, we designed and synthesized a new dihydroquinoline, namely TQ‐H_2_ for targeting cancer therapy. TQ‐H_2_ can specifically react with ·OH to form a cyclic hemiacetal, TQ‐HA, which generates ROS, including ·OH, self‐amplifying its presence in the tumor microenvironment (**Figure** **1**). By means of in vivo chemical transformation at the tumor site and self‐enhancement to amplify the tumor‐specific biomarker, we achieve integrated diagnosis and precise therapy overcoming prior limitations in tumor theranostics.

## 2 Results and Discussion

### 2.1 Synthesis and Characterization

We designed and synthesized a new molecule called TQ‐H_2_ following the synthetic route outlined in Scheme S1 (Supporting Information). The starting materials 1‐[4‐(*N*‐phenylanilino)phenyl]ethanone and 3‐formyl‐1‐methylquinolin‐1‐ium iodide were synthesized according to previous reports,^[^ ^41^, ^55^ ^]^ and their structures were confirmed by ^1^H and ^13^C NMR spectroscopy (Figures S1–S4, Supporting Information). TQ‐H_2_ exhibits high reactivity to ·OH, though its reaction mechanism appears distinct from previous predictions (Figure 1). Upon reacting with ·OH,^[^ ^56^, ^57^ ^]^ TQ‐H_2_ forms TQ‐HA, a cyclic hemiacetal. All the new compounds were characterized using ^1^H and ^13^C NMR, and high‐resolution mass spectroscopies with satisfactory results (Figures S5–S10, Supporting Information). Importantly, we successfully obtained the single crystal structure of TQ‐HA (Figure S11, Supporting Information), despite its poor stability and the presence of disordered solvents, resulting in a relatively high R1 value (≈13%). The accurate single‐crystal structure confirmed the reaction mechanism of TQ‐H_2_ to TQ‐HA.

### 2.2 Photophysical Property and Hydroxyl Radical Response

**Figure 2:**
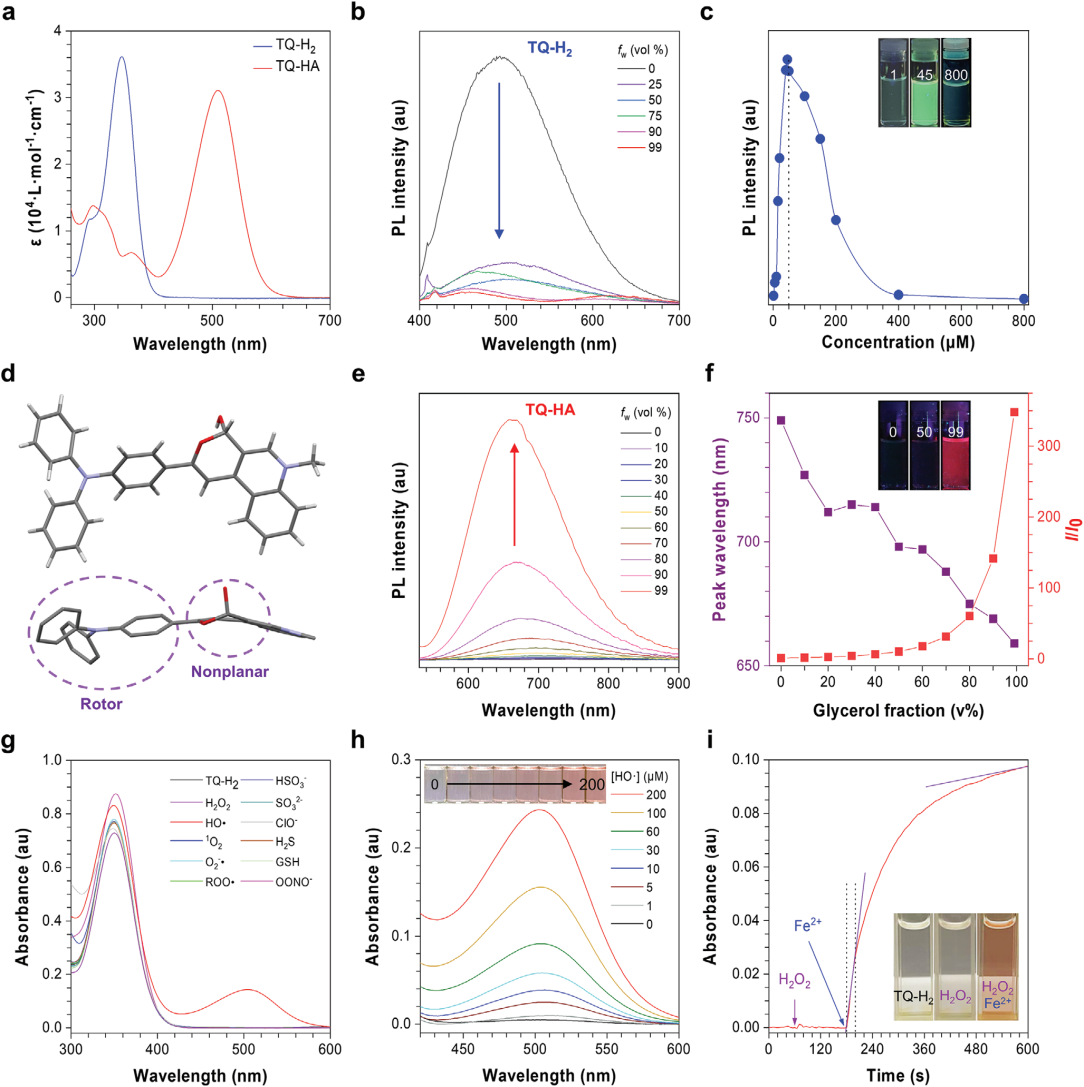
a) Absorption spectra of TQ‐H_2_ and TQ‐HA in acetonitrile. b) Photoluminescence (PL) spectra of TQ‐H_2_ (10 × 10^−6^ m) in acetone/water mixtures with different water fractions (*f*_w_), excited at 365 nm. c) Plot of PL intensity at peak maximum of TQ‐H_2_ in acetone against different solution concentrations. Excitation wavelength: 365 nm. Inset: Photos obtained under the illumination of a UV lamp (365 nm). d) Single crystal structure of TQ‐HA. e) PL spectra of TQ‐HA (10 × 10^−6^ m) in water/glycerol mixtures with different glycerol fractions (*f*_gly_). Excitation wavelength: 510 nm. f) Change in relative PL peak intensity (*I*/*I*_0_) and wavelength of TQ‐HA in different water/glycerol mixtures with different *f*_gly_, where *I*_0_ was the PL peak intensity in water solution. g) Absorption spectra of TQ‐H_2_ (20 × 10^−6^ m, ACN/H_2_O = 4/1 v/v) in the presence of various analytes. ACN = acetonitrile, [·OH] = 50 × 10^−6^ m, [OONO^−^] = 100 × 10^−6^ m, [others] = 500 × 10^−6^ m. h) Absorption spectra of TQ‐H_2_ (20 × 10^−6^ m, ACN/H_2_O = 4/1 v/v) after reaction with ·OH with different concentrations. i) Kinetic measurement of the absorption intensity change of TQ‐H_2_ at 500 nm (20 × 10^−6^ m, ACN/H_2_O = 4/1 v/v) upon reaction with ·OH (200 × 10^−6^ m). Inset: Photos in (c,f) were obtained under the illumination of a 365&amp;#x000A0;nm UV lamp. Photos in (h,i) were obtained under indoor white light.

TQ‐H_2_ absorbs light at 346 nm, while TQ‐HA shows redder absorption at 510 nm, which is consistent with the corresponding simulation calculations (**Figure** **2**a, Figures S12 and S13, Supporting Information). Electron density separation in the highest occupied and lowest unoccupied molecular orbitals of TQ‐HA results in a reduced bandgap (2.18 eV), compared to TQ‐H_2_ (3.82 eV). The hydrophobic TQ‐H_2_ (CLogP: 7.56) shows strong cyan fluorescence at 510 nm when molecularly dissolved in a good solvent such as acetone. However, upon adding a poor solvent such as water, the cyan fluorescence is quenched at high water content, probably due to the aggregate formation (Figure 2b). We also tested its fluorescence spectrum at different concentrations. As shown in Figure 2c and Figure S14 (Supporting Information), as the concentration increases, the fluorescence intensity increases initially and then decreases rapidly at concentrations exceeding 400 × 10^−6^ m. The single crystal analysis of TQ‐HA reveals a hemicyclic acetal structure with electron‐donating triphenylamine and electron‐withdrawing dihydroquinoline groups (Figure 2d). Same as the absorption, the fluorescence of the TQ‐HA red‐shift is caused by the electronic communication between the electron donor and the electron acceptor. Unlike TQ‐H_2_, the amphiphilic TQ‐HA (CLogP: 1.29) emits no fluorescence in water/ACN (99/1, v/v), but strong deep red fluorescence at 660 nm in glycerol/water mixtures with high glycerol contents (Figure 2e,f). TQ‐HA also exhibitsNIR emission at 710 nm upon aggregation in diethyl ether/ACN mixtures (Figure S15, Supporting Information), demonstrating its AIE activity. Due to its nonplanar cyclic hemiacetal structure with a dihedral angle of more than 50° and free rotating benzene rings in the triphenylamine group (Figure 2d and Figure S11, Supporting Information), TQ‐HA avoids fluorescence quenching in the aggregate state and activates AIE in high viscosity environments where molecular motion is restricted (Figure S16, Supporting Information). Clearly, TQ‐H_2_ is an ACQ molecule, but TQ‐HA is an AIE luminogen (Table S1, Supporting Information).

The significant difference in the absorption of TQ‐H_2_ and TQ‐HA enables selective detection of the chemical transformation. While TQ‐H_2_ shows no appreciable absorption changes in the presence of common reactive oxygen and nitrogen species (RONS), its absorption significantly red‐shifts to 500 nm in the presence of ·OH, confirming its specific reactivity (Figure 2g). TQ‐H_2_ is highly sensitive to ·OH, responding even at 1 × 10^−6^ m concentrations in a dose‐dependent manner (Figure 2h). As the ·OH concentration increases, the TQ‐H_2_ solution gradually changes from colorless to red, allowing the naked‐eye detection. Tracking absorption at 500 nm reveals that TQ‐H_2_ remains stable in the presence of hydrogen peroxide (Figure 2i). However, upon adding ferrous ions, the Fenton reaction generates ·OH,^[^ ^57^ ^]^ leading to a rapid 260‐fold increase in absorption within 20 seconds, accompanied by a distinct color change. These results demonstrate that TQ‐H_2_ is an excellent ·OH probe with high specificity, super sensitivity, and fast response.

### 2.3 ROS Generation

**Figure 3:**
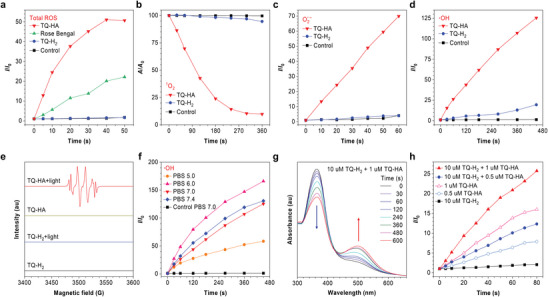
Time–course plots of a) DCFH fluorescence enhancement, b) ABDA decomposition, c) DHR123 fluorescence enhancement, and d) HPF fluorescence enhancement in the presence of TQ‐H_2_ (10 × 10^−6^ m) or TQ‐HA (10 × 10^−6^ m) in pH 7.0 PBS solution with 1 vol% DMSO under white light irradiation (5 mW cm^−2^). [Rose Bengal] = 10 × 10^−6^ m, [DCFH] = 50 × 10^−6^ m, [ABDA] = 50 × 10^−6^ m, [DHR123] = 20 × 10^−6^ m, [HPF] = 10 × 10^−6^ m. e) ESR signals of DMPO radical (superoxide and hydroxyl) adducts in the presence of TQ‐H_2_ or TQ‐HA in pH 7.0 PBS before and after white light irradiation. f) Time–course plots of HPF fluorescence enhancement in the presence of TQ‐HA in different PBS solution with 1 vol % DMSO under white light irradiation (5 mW cm^−2^). g) Absorption spectra of TQ‐H_2_ (10 × 10^−6^ m) and TQ‐HA (1 × 10^−6^ m) in 7.0 PBS solution with 2 vol% DMSO under white light irradiation (5 mW cm^−2^) at different time. h) Time‐course plots of DCFH fluorescence enhancement in the presence of different PSs in pH 7.0 PBS solution with 1 vol% DMSO under white light irradiation (5 mW cm^−2^).

We further explored the ROS generation capability of TQ‐H_2_ and TQ‐HA using non‐fluorescent 2′,7′‐dichlorodihydrofluorescein (DCFH) as an indicator. As shown in **Figures** **3**a and S17 (Supporting Information), TQ‐HA triggers a large fluorescence enhancement of DCFH, indicative of its strong ROS generation ability. Indeed, TQ‐HA can quickly generate a large amount of ROS under weak white light irradiation (5 mW cm^−2^) within 40 s. In contrast, TQ‐H_2_ did not generate detectable ROS under the same conditions. To identify the type of ROS produced by TQ‐HA, we employed three additional indicators, namely 9′,10′‐anthracenediyl‐bis(methylene)‐dimalonic acid (ABDA), dihydrorhodamine 123 (DHR123) and hydroxyphenyl fluorescein (HPF), which detect the presence of singlet oxygen (^1^O_2_), oxynegative radical ions (O_2_^·−^) and ·OH, respectively. As depicted in Figure 3b–d and Figures S18–S20 (Supporting Information), TQ‐HA rapidly generated ROS, including ^1^O_2_ (Type II), O_2_^·−^ (Type I), and ·OH (Type I), under the white light irradiation. This finding was confirmed by electron spin resonance (ESR) spectroscopy, which revealed a distinct DMPO‐OH· signal peak, indicating hydroxyl radical generation (Figure 3e). The efficacy of TQ‐HA in generating either Type I or Type II ROS was demonstrated across a physiological pH range (5.0‐7.4), highlighting its potential for intracellular application (Figure 3f and Figures S21 and S22, Supporting Information). These results indicate that TQ‐H_2_ does not generate ROS before reaction with ·OH, whereas the generated product TQ‐HA is a strong ROS generator with potential PDT application.

Interestingly, adding a small quantity of TQ‐HA (1:10 mol ratio) to the TQ‐H_2_ solution results in a significant enhancement in the intensity of TQ‐HA's characteristic absorption at 500 nm after white light irradiation, while the corresponding absorption at 350 nm (for TQ‐H_2_) decreased markedly (Figure 3g). This indicates that TQ‐H_2_ was undergoing a gradual conversion to TQ‐HA under light, which is a light‐controlled autocatalytic chemical reaction.

**Figure 4:**
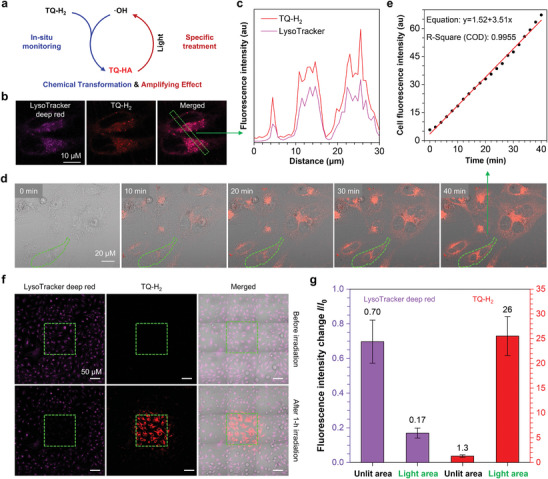
a) Schematic diagram of chemical transformation of TQ‐H_2_ and amplifying effect of TQ‐HA. b) CLSM images of HeLa cells stained with TQ‐H_2_ (5 × 10^−6^ m) without washing and sequential scanning. LysoTracker deep red with lysosomes appeared in violet color. c) Quantitative analysis of colocalization using ZEISS ZEN and Image J. d) CLSM images of real‐time monitoring HeLa cells stained with TQ‐H_2_ (5 × 10^−6^ m) without washing. The sequential scanning was performed every 2 min by using a 488 nm laser (2% power). e) Quantitative relationship between the photoluminescence (PL) intensity of cell and monitoring time. f) CLSM images of HeLa cells stained with LysoTracker deep red (250 × 10^−9^ m) and TQ‐H_2_ (5 × 10^−6^ m) without washing and the sequential scanning of the center area (green box) by using a 488 nm laser with 1% power for 1 h. g) Change of relative PL intensity (*I*/*I*_0_) of LysoTracker deep red and TQ‐HA in the unlit and irradiated areas, where *I*_0_ was the PL intensity in the absence of light irradiation (mean ± SD, *N* = 4).

This autocatalytic reaction results in a significant amplification of ROS generation. As illustrated in Figure 3h and Figure S23 (Supporting Information), the fluorescence increase of DCFH was used to measure ROS generation. TQ‐H_2_ alone did not produce ROS, but the addition of a small amount of TQ‐HA (0.5 × 10^−6^ m) to TQ‐H_2_ (10 × 10^−6^ m) markedly increased DCFH fluorescence by 56.9% over TQ‐HA alone within 80 s. Increasing TQ‐HA to 1 × 10^−6^ m further amplified the signal, with a 60.9% increase compared to TQ‐HA alone. The combined analysis of Figure 3g and the aforementioned results suggests that the presence of TQ‐HA can effectively promote the conversion of TQ‐H_2_ to TQ‐HA, thereby further increasing ROS generation under white light irradiation, resulting in a self‐recycling system. As shown in **Figure** **4**a, the ·OH produced by TQ‐HA reacts with TQ‐H_2_, driving further TQ‐HA formation. This establishes a photo‐controlled self‐enhancement system, which is expected to realize the amplification effect of PDT.

### 2.4 Monitoring Intracellular Hydroxyl Radical

Chemical experiments have demonstrated that TQ‐H_2_ is an excellent probe for ·OH detection, and undergoes chemical transformation with ·OH to generate hemiacetal TQ‐HA (Figure 4a). Dynamic light scattering (DLS) analysis suggests that TQ‐H_2_ can form nanoaggregates with a size of ≈140 nm in the water suitable for cell entry (Figure S24, Supporting Information). As illustrated in Figures S25 and S26 (Supporting Information), TQ‐H_2_ and TQ‐HA exhibit low cytotoxicity in both HeLa tumor cells and LO2 normal cells in the absence of light. Upon irradiation with white light, the half‐maximal inhibitory concentration (IC50) of TQ‐H_2_ and TQ‐HA for HeLa cells was 13.7 × 10^−6^ and 10.6 × 10^−6^ m, respectively, while for LO2 cells, it was 17.5 × 10^−6^ and 9.6 × 10^−6^ m, respectively. The lower IC50 in HeLa cells indicates a moderate selectivity for tumor cells, likely due to higher baseline levels of ·OH in tumors. We next evaluated intracellular localization and reactivity. As shown in Figure 4b, we stained the cells with a lysosome probe called LysoTracker deep red. After washing, the cells were co‐stained with TQ‐H_2_ without washing followed by imaging in real time. Confocal microscopy imaging showed that TQ‐H_2_ colocalized with the lysosomes, with a Pearson correlation coefficient of 0.90 after 1 h of staining (Figure S27, Supporting Information). The fluorescence intensity distributions of the two probes at different positions are also completely overlapped (Figure 4c). These results indicate that TQ‐H_2_ preferentially undergoes chemical transformation in lysosomes, probably due to the presence of more ·OH and H^+^ in this organelle.

We further investigated real‐time imaging of intracellular ·OH using TQ‐H_2_. As shown in Figure 4a,d, TQ‐H_2_ reacts with ·OH, and the resulting TQ‐HA binds to and illuminates the cells, typical of positively charged amphiphilic AIE dyes. In comparison to the culture medium, the viscosity within the organelle is high, for example, lysosomal viscosity can range from 50 to 90 cP.^[^ ^58^ ^]^ Due to the restriction of intramolecular motion by the biological binding, the fluorescence of TQ‐HA was activated, and the fluorescence intensity increased gradually over time. Quantitative analysis indicates a good and linear relationship between the intracellular fluorescence intensity and time within 40 min with a R‐square value of 0.9955 (Figure 4e), confirming that TQ‐H_2_ can quantitatively detect ·OH in living cells. Commercial probes like LysoTracker Deep Red suffer from poor photostability and rapid photobleaching, with fluorescence decaying to 17% within 1 h of continuous 488 nm laser exposure (Figure 4f,g). Such properly makes the probe almost impossible to complete the continuous labeling and monitoring because the SNR drops severely. In contrast, TQ‐H_2_ is a lysosome‐targeted turn‐on probe. Under continuous light stimulation, ROS, including ·OH, are generated, and the red fluorescence signal strengthens over time, consistent with the self‐amplification observed in Figure 3g,h. After 1 h, the fluorescence intensity of light area was enhanced by 26 times. We successfully recorded a 90 min video to demonstrate the change of ·OH in living cells using TQ‐H_2_ (Video S1, Supporting Information).

### 2.5 Inducing Apoptosis and Ferroptosis

**Figure 5:**
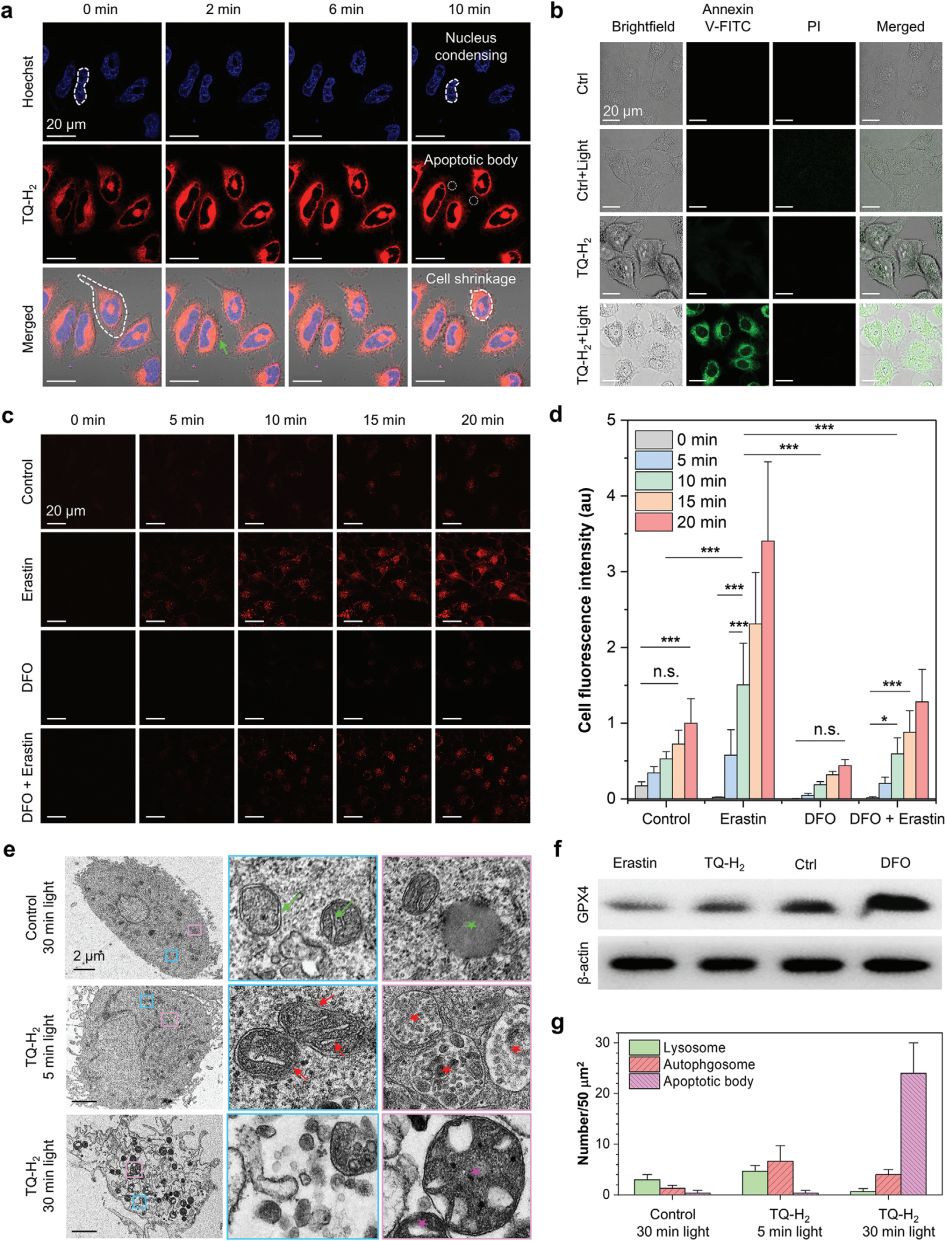
a) CLSM images of HeLa cells stained with TQ‐H_2_ (10 × 10^−6^ m) for 10 min under 405 nm laser (2.5% power) stimulation at different time. Hoechst with blue fluorescence was used to stain the nucleus. b) CLSM images of annexin V‐FITC/PI double‐stained HeLa cells after different treatments. Annexin V‐FITC was visualized by a green signal, and PI was visualized by a red signal. (c) Fluorescence images of HeLa cells stained with TQ‐H_2_ (5 × 10^−6^ m) under different conditions. d) Quantitative analysis of cell fluorescence intensity in (c) (mean ± SD, *N* = 10). The statistical significance in this study is determined by a one‐tailed t‐test at *p < 0.05, **p < 0.01, ***p < 0.001. OriginPro 2021 was used for statistical analysis. e) TEM images after different treatments. The green triangle arrow points to the normal mitochondrial morphology, the red triangle arrows show the damaged mitochondria morphology, the green stars represent the lysosome, the red stars indicate the autophagosome‐like vesicles, and the purple stars point to the autophagosome‐like vesicles. f) Western blot detection of GPX4. g) Quantitative statistics of the autophagosome‐like vesicles after different treatments (mean ± SD, *N* = 3).

As shown in Figure 4a, TQ‐H_2_’s detection and generation of ·OH enable effective monitoring and promotion of cell death by chemical transformation and self‐amplifying. Long‐term live cell imaging revealed significant cell death in illuminated areas compared to non‐illuminated areas (Figure 4f and Figure S28, Supporting Information), suggesting that TQ‐H_2_ converts to TQ‐HA, which generates large amounts of ROS under light irradiation, efficiently inducing the cell death. Real‐time confocal microscopy imaging of TQ‐H_2_‐stained HeLa cells (**Figure** **5**a) showed that this probe not only monitors intracellular ·OH in real time but also induces apoptosis through ROS generation. After just 10 min of light irradiation, hallmark features of apoptosis appeared: nuclear condensation, cell shrinkage, and apoptotic body release. The apoptotic bodies, stained red (Figure S29, Supporting Information), suggest that TQ‐HA is released from lysosomes after cell death, staining cell membranes due to high membrane viscosity. We confirmed apoptosis by annexin V‐FITC/PI double fluorescence staining (Figure 5b).^[^ ^35^, ^47^ ^]^ Only the TQ‐H_2_+Light group shows obvious annexin V‐FITC (early apoptosis indicator) fluorescence, while no fluorescence signals from PI (late apoptosis indicator) are detected, indicating early apoptosis. By taking the advantage of the probe's long‐term tracking capability, we reduced the concentration of the probe and the laser power and successfully revealed the dynamic process of apoptosis induced by TQ‐H_2_ in real‐time (Video S2 and Figure S30, Supporting Information). Control groups without TQ‐H_2_ showed no significant cell death or apoptosis features during the 2 h observation period (Figures S31 and S32, Supporting Information).

Ferroptosis is accompanied with ·OH generation because ·OH plays a key role in lipid peroxidation.^[^ ^35^, ^36^, ^59^ ^]^ As shown in Figure 5c,d, red fluorescence signals were detected in TQ‐H_2_‐stained cells treated with Erastin (ferroptosis activator) within 5 min, while the control and the deferoxamine (DFO, ferroptosis inhibitor) groups showed no significant change. At 10 min, significant differences (***p < 0.001) are observed in all the investigated groups. After 20 min, the average fluorescence intensity of the cells in the activation group was 3.4 times and 7.7 times higher than the control group and the inhibition group, respectively. These results indicate that TQ‐H_2_ is an excellent ferroptosis probe. Transmission electron microscopy (TEM) imaging further confirmed apoptosis and ferroptosis. As shown in Figure 5e, the bilayer membrane structure and the cristae structure of mitochondria are severely damaged after PDT for 5 min. After 30 min, the mitochondrial structure was completely destroyed. Through the Western blot experiments, we found that the expression of GPX4 protein, a pivotal indicator of ferroptosis,^[^ ^60^ ^]^ was significantly reduced during the cell death (Figure 5f). Ferroptosis‐related genes (ACSL4, PTGS2, NRF2, GPX4) were also analyzed via RT‐qPCR, with results similar to the Erastin group, confirming ferroptosis (Figure S33, Supporting Information).

Additionally, we counted the number of autophagosome‐like vesicles and found that the value increases with time. The number of autophagosomes first increased and then decreased, while apoptotic body numbers rose sharply (Figure 5g). Combining with the fact that the transformation of the probe occurs in the lysosome, this statistical result suggests that the cells also undergo lysosome‐dependent death. TQ‐H_2_ not only tracks ·OH in lysosomes but also triggers apoptosis, ferroptosis, and lysosome‐dependent death through chemical transformation and self‐amplification.

### 2.6 In Vivo Theranostics

**Figure 6:**
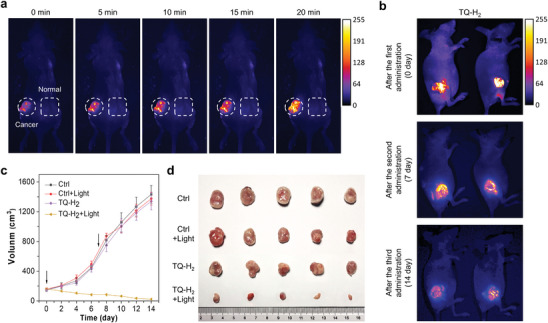
a) The tumor section‐specific self‐activation property of TQ‐H_2_. TQ‐H_2_ loaded mPEG‐b‐PLA micelles were sprayed on the tumor and opposite skin surface at a dosage about 10 mg kg^−1^. The micelles left on the surface were wiped off after 12 h. Then, the tumor was irradiated using a white light laser array (5 mW cm^−2^) as the indicated time. Imaging was performed after each light irradiation. Excitation wavelength = 505 nm, emission wavelength = 650 nm. The circles and rectangles in the image indicated the location of tumors and skin sprayed TQ‐H_2_, respectively. b) Fluorescence images of TQ‐H_2_ + light treatment groups at different time. Excitation wavelength = 505 nm, emission wavelength = 650 nm. c) Graphs of tumor volume of nude mice (mean ± SEM, *N* = 5) after treatment with TQ‐H_2_ (10 mg kg^−1^), and physiological saline with or without light irradiation using a white light laser array (12 J cm^−2^). Performing transdermal application of micelles of TQ‐H_2_ for every 7 d, and the light irradiation was performed for 12 h after drug administration. The black arrows indicated the time point for drug administration. d) Tumors separated from nude mice.

To validate the therapeutic effect in vivo, polymeric micelles of TQ‐H_2_ were constructed using mPEG‐b‐PLA (Figure S34, Supporting Information). TEM analysis confirmed spherical nanoparticles, and DLS measurements revealed a stable size of 139 nm (Z‐average, PDI = 0.118) over 72 h. The micelles exhibited a drug loading rate of 77.4%, as determined by UV–Vis absorption. Anticancer experiments are performed in a xenograft model using BALB/C nude female mice bearing HeLa tumors (Figure 1). The mice are randomly divided into 4 groups (Control, Control+light, TQ‐H_2_, TQ‐H_2_+light). TQ‐H_2_ micelles were administrated through tumor localized transdermal.^[^ ^61^ ^]^ Tumors were impregnated with the micelles for 12 h, and then wiped off using a 75% alcohol pad. The light irradiation source was a white light array (12 J cm^−2^). The tumor volume and the mouse weight are routinely monitored for 14 d. Interestingly, as shown in **Figures** **6**a and S35 (Supporting Information), strong fluorescence was observed in the tumors, while little or no fluorescence appeared at control sites or other organs. This demonstrates that TQ‐H_2_ specifically targets the tumor microenvironment and is activated to form TQ‐HA within 12 h, confirming its tumor‐targeting capability. Moreover, under white light irradiation, fluorescence intensity at the tumor site gradually enhanced by the self‐amplifying to allow clear visualization of tumor and consistently enriched in the tumor site during the two weeks of treatment (Figure 6a,b). Compared with the control groups, TQ‐H_2_ alone shows only a low inhibitory effect on the tumor growth. However, the growth of tumors was significantly inhibited upon light irradiation (Figure 6c,d). At the end of treatment, the tumors treated with TQ‐H_2_+light show a large number of apoptotic tumor cells than other groups (Figure S36, Supporting Information), and the tumor volume decreases by about 98% compared with the control group (Figure 6c,d). Moreover, no significant change in the weight of mice or serious structural and pathological organ alternation are detected in the TQ‐H_2_ treated group (Figures S37–S39, Supporting Information), indicating that TQ‐H_2_ and its light‐activated form cause no obvious side effects.

This study revealed a probe TQ‐H_2_ capable of responding to the cellular or tumor microenvironment, achieving in situ self‐amplification and efficient PDT effects both in vitro and in vivo. This research builds on previous work by proposing a novel mechanism of dihydroquinoline and hydroxyl radical reactions, offering a new approach to integrated tumor diagnosis and therapy based on microenvironment‐dependent chemical transformation and self‐amplification.

TQ‐H_2_ is an ACQ molecule while TQ‐HA exhibits the AIE activity with certain water solubility. TQ‐H_2_ shows no fluorescence in the biological environment of cell but after chemical conversion with ·OH in the lysosome, TQ‐HA with strong red emission will be generated. This enables wash‐free bioimaging and real‐time monitoring. Excitingly, TQ‐HA also has strong ROS generation capacity, especially ·OH, under light irradiation, creating a cyclic amplification loop that promotes further TQ‐HA formation. The accumulated ROS induces multiple cell death pathways, which we observed in prolonged experiments showing apoptotic signaling (Video S2, Supporting Information). Initially, TQ‐HA is generated in lysosomes, leading to lysosome‐dependent cell death. High intracellular ·OH concentrations also trigger ferroptosis. We hypothesize that apoptosis is the primary mode of death, while ferroptosis and lysosome‐dependent death occur as secondary processes, reinforcing apoptosis.

In Figure 4b, we realized that the probe lights up lysosomes, suggesting two potential mechanisms: 1) TQ‐H_2_ distributes across the cell, with lysosomes providing the most suitable microenvironment for chemical transformation, or 2) the probe specifically targets lysosomes. We found that the fluorescence intensity of cells shows a linear relationship with time (Figure 4e). An implication of this finding is that the probe aggregates enter the cell at a uniform speed in the first 40 min. This is in conjunction with the assumption that the cell is in a steady state, where the production of hydroxyl radicals is also linear. The Pearson correlation value for colocalization (Figure S27, Supporting Information) depends on two factors: TQ‐HA and LysoTracker Deep Red targeting the same sites, and the accumulation of TQ‐HA in lysosomes. Colocalization increases linearly from 0 to 40 min, followed by gradual saturation between 40 and 70 min. After 70 min, the TQ‐HA signal exceeds that of LysoTracker Deep Red, leading to a decrease in colocalization values.

According to the existing results, we can preliminarily believe that the first step of chemical transformation occurs in lysosomes. Once cells undergo death and lysosomal rupture, TQ‐HA can bind to polysaccharides, glycoproteins, and proteins throughout the cell via electrostatic or amphiphilic interactions, inducing multiple cell deaths. Figure 5 further demonstrates that this chemical transformation and self‐amplification enable real‐time ·OH imaging and induce ROS‐driven cell death. Lysosomes, cytoplasm, and cell membranes are all affected, leading to rapid cell death, providing a promising approach for future animal treatments. TQ‐H_2_ demonstrates excellent tumor site‐specificity in vivo primarily due to its specific response to ·OH in the tumor microenvironment. This facilitates the precise conversion of TQ‐H_2_ to TQ‐HA at the tumor site. Additionally, the photo‐controlled amplification effect promotes the in situ enhancement of TQ‐HA, leading to effective in vivo PDT. Moreover, TQ‐HA are positively charged amphiphilic molecules that can interact with tumor cells through electrostatic or amphiphilic interactions. Furthermore, the special structure of cyclic hemiacetal enables acetal or hemiaminal reactions with hydroxyl or amino groups in hydrophobic environments such as cell membranes, proteins, and lipid droplets. These potential physical effects and chemical reactions may increase the residence time at the tumor site, thereby enhancing the effectiveness of the treatment.

Based on the response principle, TQ‐H_2_ will have more pronounced imaging and therapeutic effects on tumors with high hydroxyl radical expression. In this work, we used a subcutaneous tumor model in which the therapeutic light was white light. Our system may therefore be more effective in treating superficial tumors such as skin cancer. By developing NIR‐II probes with the same mechanism, or by delivering light into the body using optical fibers, together with photodynamic therapy, it will be possible to treat deep tumors.

## 3 Conclusion

In summary, we developed a novel probe (TQ‐H_2_) with high specificity, super sensitivity, and fast response to ·OH for both in vitro and in vivo theranostics. TQ‐H_2_ targets lysosomes and undergoes a chemical transformation with ·OH to produce TQ‐HA, which emits strong red fluorescence in high‐viscosity environments. TQ‐HA also possesses robust ·OH generation capabilities under light irradiation, enabling self‐amplification of the chemical transformation and accumulating more TQ‐HA. This light‐controlled autocatalytic reaction enhances the contrast between tumor and normal tissues while increasing ROS production. In vitro, TQ‐H_2_ achieves the monitoring and induction of a variety of cell death pathways, including apoptosis, ferroptosis and lysosome‐dependent death. In vivo experiments demonstrate that TQ‐H_2_ exhibits excellent tumor specificity and PDT effect, leading to effective tumor treatment. Based on its unique response principle, TQ‐H_2_ is expected to offer more pronounced imaging and therapeutic effects in tumors with high ·OH expression. The discovery of a microenvironment‐responsive and self‐amplifying probe, together with its potential for specific diagnosis and treatment of tumors, represents a significant advance in the field of chemical biology and holds great promise for the design of intelligent probes for various biological signals.

## 4 Experimental Section

### Materials

Triphenylamine (98%, Energy Chemical), ZnCl_2_ (98%, Energy Chemical), ethanoyl chloride (98%, Energy Chemical), anhydrous Na_2_SO_4_ (AR, Dieckmann), quinoline‐3‐carbaldehyde (98%, Dieckmann), methyl iodide (99.9%, Energy Chemical), FeCl_2_·4H_2_O (99%, Sigma‐Aldrich), 2,3,5,6‐tetrachlorocyclohexa‐2,5‐diene‐1,4‐dione (98%, Sigma‐Aladdin), hydrogen peroxide (30 wt% solution in water, Fujifilm Wako), 2′,7′‐dichlorodihydrofluorescein diacetate (DCFH‐DA, ≥95, Sigma‐Aldrich), 9,10‐anthracenediyl‐bis(methylene)dimalonic acid (ABDA, 97%, Leyan), dihydrorhodamine 123 (DHR 123, ≥95%, Sigma‐Aldrich), hydroxyphenyl fluorescein (HPF, 5 × 10^−3^ m in DMF, Sigma‐Aldrich), and mPEG‐b‐PLA (2k‐5k, Sigma‐Aldrich) were purchased. Other reagents were purchased from Sigma‐Aldrich and used as received.

### Synthesis of 1‐[4‐(N‐Phenylanilino)phenyl]ethenone

Triphenylamine (8.84 g, 36 mmol), ZnCl_2_ (4.90 g, 36 mmol), and anhydrous dichloromethane (DCM, 80 mL) were added to a round‐bottom flask. The mixture was stirred at room temperature, and ethanoyl chloride (2.84 g, 36 mmol) was slowly added. The color of the solution gradually changed from yellow to dark green. After refluxing overnight under nitrogen at 40 °C, the solution darkened further. Once cooled to room temperature, dilute hydrochloric acid was added, and the mixture was stirred before separating the layers. The organic phase was collected, washed with water three times, dried with anhydrous Na_2_SO_4_, and concentrated by rotary evaporation. The crude product was purified by silica‐gel column chromatography, yielding a pale yellow product (80%). ^1^H NMR (400 MHz, CDCl_3_): *δ* 7.84–7.75 (m, 2H), 7.37–7.29 (m, 4H), 7.18–7.10 (m, 6H), 7.02–6.96 (m, 2H), 2.53 (s, 3H). ^13^C NMR (100 MHz, CDCl_3_): *δ* 195.66, 151.29, 145.61, 129.02, 128.88, 128.74, 125.09, 123.76, 118.79, 25.41.

### Synthesis of 3‐Formyl‐1‐methylquinolin‐1‐ium iodide

Quinoline‐3‐carbaldehyde (1.57 g, 10 mmol), methyl iodide (14.2 g, 100 mmol), and 15 mL acetonitrile were added to a flask, then heated to reflux (50‐60 °C) under nitrogen protection overnight. After cooling to room temperature, an orange‐yellow precipitate formed, which was collected by filtration and washed with ACN/DCM and dried in a vacuum. The resulting 3‐formyl‐1‐methylquinolin‐1‐ium iodide was obtained as an orange‐yellow solid (yield: over 95%). ^1^H NMR (400 MHz, DMSO‐d_6_): *δ* 10.30 (s, 1H), 10.01 (dd, *J* = 1.7, 0.8 Hz, 1H), 9.83 (t, *J* = 1.3 Hz, 1H), 8.69 (dd, *J* = 8.3, 1.5 Hz, 1H), 8.61 (dd, *J* = 8.8, 1.0 Hz, 1H), 8.45 (ddd, *J* = 8.8, 7.0, 1.5 Hz, 1H), 8.18 (ddd, *J* = 8.1, 7.0, 0.9 Hz, 1H), 4.73 (s, 3H). ^13^C NMR (100 MHz, DMSO‐d_6_): *δ* 189.01, 149.95, 148.68, 139.57, 137.94, 132.17, 130.90, 128.91, 128.47, 119.69, 45.75.

### Synthesis of TQ‐H_2_

1‐[4‐(Diphenylamino)phenyl]ethan‐1‐one (574 mg, 2 mmol), 3‐formyl‐1‐methylquinolin‐1‐ium iodide (299 mg, 2.4 mmol), 30 mL MeOH, and 30 mL H_2_O were added into a spherical flask. Then NaOH (160 mg, 4 mmol) was added under nitrogen. The mixture was heated and stirred overnight. After cooling, the mixture was extracted with DCM, and the organic phases were combined, washed with water, dried with anhydrous Na_2_SO_4_, and concentrated. The product was purified by silica‐gel column chromatography yielding a bright yellow powder (30%). ^1^H NMR (400 MHz, CD_3_CN): *δ* 9.12 (s, 1H), 7.75–7.65 (m, 2H), 7.44–7.29 (m, 4H), 7.23–7.06 (m, 9H), 7.00–6.92 (m, 2H), 6.86–6.78 (m, 2H), 4.55 (t, *J* = 6.0 Hz, 1H), 3.34 (s, 3H), 3.01 (d, *J* = 6.0 Hz, 2H). ^13^C NMR (100 MHz, CD_3_CN): *δ* 197.65, 187.96, 152.99, 152.92, 147.45, 139.51, 130.78, 130.73, 130.49, 128.43, 127.51, 127.09, 125.88, 124.79, 119.83, 115.60, 114.37, 47.85, 39.64, 33.02. HRMS: 481.1890 [C_31_H_26_N_2_O_2_+Na]^+^.

### Synthesis of TQ‐HA

TQ‐H_2_ (900 mg, 2 mmol), 2,3,5,6‐tetrachlorocyclohexa‐2,5‐diene‐1,4‐dione (246 mg, 10 mmol), 100 mL ACN were thoroughly mixed. While stirring, 1.0 mL 30% H_2_O_2_ solution was slowly added, and the color of the solution rapidly changed to dark red. The mixture was stirred at room temperature for 2 h. After solvent removal by rotary evaporation, the crude product was purified by silica‐gel column chromatography, yielding a deep red powder (95%). ^1^H NMR (400 MHz, DMSO‐*d*_6_): *δ* 9.08 (d, *J* = 89.3 Hz, 2H), 8.56 (s, 1H), 8.30 (s, 1H), 8.18 (s, 1H), 8.06 (s, 2H), 7.97 (s, 1H), 7.82 (s, 1H), 7.40 (s, 4H), 7.16 (s, 6H), 6.90 (d, *J* = 47.9 Hz, 3H), 4.41 (s, 3H). ^13^C NMR (100 MHz, CD_3_CN/D_2_O) *δ* 163.35, 151.74, 145.92, 144.85, 144.80, 138.48, 135.20, 130.18, 129.93, 129.16, 128.86, 126.22, 126.18, 125.33, 123.53, 122.56, 119.29, 118.72, 116.82, 92.80, 91.18, 45.06. HRMS: 457.1911 [C_31_H_25_N_2_O_2_]^+^. The CCDC deposition number of TQ‐HA crystal is 2352472.

### Hydroxyl Radical Detection

Quantitative hydroxyl radicals were produced by the Fenton reaction of ferrous ions and hydrogen peroxide. For example, 10 × 10^−6^ m HO· was generated by mixing 100 × 10^−6^ M H_2_O_2_ with 10 × 10^−6^ M Fe^2+^.

### ROS Detection

Various ROS indicators were used: DCFH for general ROS, ABDA for singlet oxygen, DHR123 for superoxide anions, and HPF for hydroxyl radicals. ROS indicators were added to TQ‐H_2_ or TQ‐HA solutions, followed by white light irradiation. Fluorescence or absorption changes were monitored to reflect ROS generation. Test parameters: for DCFH and DHR123, excitation = 480 nm, emission = 526 nm; for HPF, excitation = 480 nm, emission = 514 nm; for ABDA, recorded absorption = 380 nm.

### Electron Spin Resonance (ESR)

HO·generation was identified on an ESR spectrometer system (Bruker, German) using DMPO as the spin trap with a microwave bridge (receiver gain, 1 × 10^5^; modulation amplitude, 1 G; scan width, 100 G; microwave power, 20 mW; modulation frequency, 100 kHz). TQ‐H_2_ (5 × 10^−6^ m) and TQ‐HA (5 × 10^−6^ m), DMPO (10 × 10^−3^ m) were mixed in PBS buffer solutions with or without light irradiation (white laser arrays, 5 mW cm^−2^, 5 min) before measurements.

### Cell Culture

HeLa cells were cultured in DMEM medium containing 10% FBS and antibiotics (100 units mL^−1^ penicillin and 100 µg mL^−1^ streptomycin) in a humidified incubator with 5% CO_2_ at 37 °C. HeLa cells were seeded in a confocal dish and incubated at 37 °C for 24 h before imaging.

### Confocal Colocalization

HeLa cells were first treated with LysoTracker deep red (250 × 10^−9^ m) for 5 min, washed three times with PBS, and then stained with TQ‐H_2_ (5 × 10^−6^ m). Confocal laser scanning microscopy (CLSM; Zeiss LSM800) was used for imaging without further washing. LysoTracker Deep Red was excited at 640 nm, with emission captured in the 650–700 nm range, while TQ‐H_2_ or TQ‐HA was excited at 488 nm, with emission collected in the 560–650 nm range.

### Monitoring Intracellular Hydroxyl Radicals

HeLa cells stained with TQ‐H_2_ (5 × 10^−6^ m) without washing were monitored in real‐time using CLSM. Sequential scans were performed every 2 min with an excitation wavelength of 488 nm (2% laser power) and an emission filter of 560–650 nm. The frame time was 2.53 s.

### Apoptosis Induction

HeLa cells were first incubated with Hoechst 33258 to stain the nucleus. After washing three times with PBS, the cells were stained with TQ‐H_2_ (10 × 10^−6^ m) for 20 min. Cells were then imaged by CLSM under 405 nm laser stimulation (2.5% power). For Hoechst, the emission was collected using a 400–470 nm filter, and for TQ‐HA, a 560–650 nm filter was used.

### Apoptosis Detection

HeLa cells were treated with fresh medium containing 10 × 10^−6^ m TQ‐H_2_ for 20 min. For the photodynamic therapy, cells were irradiated using a white light laser array (5 mW cm^−2^) for 10 min. After irradiation, cells were stained with the Annexin V‐FITC/PI Apoptosis Detection Kit (Beyotime) for 20 min (Annexin V‐FITC: 5 × 10^−6^ m, propidium iodide: 10 × 10^−6^ m). Cells were imaged by CLSM: excitation for Annexin V‐FITC was at 488 nm (emission: 505–545 nm), and for PI, excitation was at 514 nm (emission: 590–630 nm).

### Ferroptosis Monitoring

There were four groups of experiments: the control group, the ferroptosis promotion group (Erastin), the ferroptosis suppression group (DFO) and the ferroptosis suppression before promotion group (DFO+Erastin). In the promotion group, HeLa cells were incubated with a medium containing Erastin (10 × 10^−6^ m) for 16 h, washed with PBS, and stained with TQ‐H_2_ (5 × 10^−6^ m) for real‐time CLSM monitoring. In the suppression group, cells were treated with DFO (10 × 10^−6^ m) for 18 h, followed by PBS washing and TQ‐H_2_ staining (5 × 10^−6^ m). The DFO+Erastin group included pre‐treatment with DFO, followed by Erastin.

### In Vivo Experiments

All animal procedures were approved by the Institutional Animal Care and Use Committee (IACUC) of the Chinese University of Hong Kong, Shenzhen, China (Accreditation: CUHKSZ‐AE2022004). Pathogen‐free female BALB/c nude mice (4–5 weeks old) were purchased and bred at the Guangdong Medical Laboratory Animal Center.

To establish a xenograft model, HeLa cells (1 × 10⁶) were subcutaneously injected into the right axilla of each mouse. When the tumor volumes reached about 200 mm^3^, the mice were randomly divided into six groups (5 mice per group), and TQ‐H_2_ loaded mPEG‐b‐PLA micelles solution (5 mg kg^−1^ of TQ‐H_2_ in physiological saline) or the same volume of physiological saline solution (Control) was sprayed at the surface of tumors. After 12 h, the sprayed compounds were wiped off using a 75% alcohol pad. The same treatment was performed every 7 d. The tumor growth was monitored by measuring the perpendicular diameter of the tumor using calipers every 2 d and calculated according to the formula:

(1) $$\mathrm{Tumor}\;\mathrm{volume}\;\left(V\right)=\frac{\left(\mathrm{tumor}\;\mathrm{length}\right)\times {\left(\mathrm{tumor}\;\mathrm{width}\right)}^{2}}{2}$$

At the end of therapy (14 d), the tumors were separated and the inhibition rate was calculated according to the formula:

(2) $$\mathrm{Inhibitory}\;\mathrm{rate}\;\left(\%\right)=\frac{V_{\mathrm{Control}}-V_{\mathrm{drug}}}{V_{\mathrm{Control}}}\;\times 100\%$$

### Statistical Analysis

Confocal images were processed using ZEN 3.3 software (Zeiss). For the data in Figure 4d, in the graph of the selected cells, the total intensity of the red channel was statistically acquired and divided by 10^6^ to obtain relative intensity. For Figure 4f, the fluorescence intensity of the corresponding channel was statistically acquired by selecting four square areas of the unlit or irradiated areas. The unlit area of the red channel was employed as the reference for calculating the mean ± SD (*N* = 4). For Figure 5c, fluorescence intensity per unit area was obtained using the ROI Manager function in ImageJ 1.53e software. Ten cells and five background regions were selected from each image. The corrected mean fluorescence intensity (mean ± SD, *N* = 10) was determined by subtracting the background intensity. Statistical significance was assessed using *P*‐values. In TEM images, the numbers of lysosomes, autophagosomes, and apoptotic vesicles were manually counted, and the mean ± SD (*N* = 3) was calculated. Tumor volumes in Figure 6c are presented as mean ± SEM (*N* = 5).

## Conflict of Interest

The authors declare no conflict of interest.

## Supporting information



Supporting Information

Supplemental Video 1

Supplemental Video 2

## Data Availability

The data that support the findings of this study are available in the Supporting Information of this article.
