# Supplementary material for: Tumor Site‐Specific In Vivo Theranostics Enabled by Microenvironment‐Dependent Chemical Transformation and Self‐Amplifying Effect
Source: Adv Sci (Weinh). 2024 Nov 29;12(4):2409506. doi: 10.1002/advs.202409506 (PMC11789590; doi:10.1002/advs.202409506)
Supplement: Supplementary file 1 — Supporting Information [file ADVS-12-2409506-s003.docx]

Supporting Information

Tumor site-specific in vivo theranostics enabled by microenvironment-dependent chemical transformation and self-amplifying effect

Yunfei Zuo^†^, Pei Li^†^, Wen-Jin Wang^†^, Changhuo Xu, Shuting Xu, Herman H. Y. Sung, Jianwei Sun, Guorui Jin, Weiping Wang, Ryan T. K. Kwok*, Jacky W. Y. Lam* and Ben Zhong Tang*

^†^These three authors contributed equally to this work:

Yunfei Zuo, Pei Li, and Wen-Jin Wang

*The corresponding authors:

Ryan T. K. Kwok ([chryan@ust.hk](mailto:chryan@ust.hk))

Jacky W. Y. Lam ([chjacky@ust.hk](mailto:chjacky@ust.hk))

Ben Zhong Tang ([tangbenz@cuhk.edu.cn](mailto:tangbenz@cuhk.edu.cn))

# 1. General information

^1^H and ^13^C Nuclear Magnetic Resonance (NMR) spectra were measured on a Bruker AVIII 400 or NEO 400 MHz NMR spectrometer.

High-resolution MS (HRMS) was recorded on Water MS (Waters GCT PremierTM Mass Spectrometer or Waters Xevo G2-XS TOF Mass Spectrometer).

UV-Vis spectra of the samples were measured on a Shimadzu UV-2600I spectrometer.

Fluorescence spectra of solution materials were carried out on an Edinburgh Instruments FS5 Fluorescence Spectrometer.

Fluorescence spectra of solid materials were carried out on an Edinburgh Instruments FLS980 spectrometer.

The absolute fluorescence quantum yields were characterized using a Hamamatsu quantum yield spectrometer C11347 Quantaurus QY.

A suitable TQ-HA crystal was selected and on a Bruker MetalJet D2+ with a PHOTON detector diffractometer. The crystal was kept at 100.00 K during data collection. Using Olex2^[1]^, the structure was solved with the SHELXT^[2]^ structure solution program using Intrinsic Phasing and refined with the SHELXL^[3]^ refinement package using Least Squares minimization.

1. Dolomanov, O.V., Bourhis, L.J., Gildea, R.J, Howard, J.A.K. & Puschmann, H. (2009), J. Appl. Cryst. 42, 339-341.
2. Sheldrick, G.M. (2015). Acta Cryst. A71, 3-8.
3. Sheldrick, G.M. (2015). Acta Cryst. C71, 3-8.

Confocal imaging was measured on a ZEISS LSM 800 with an Airyscan Confocal Laser Scanning Microscope.

For tumors in vivo imaging: the mice were anesthetized using respiratory anesthesia (isoflurane), and imaged using in vivo imager immediately. λ_ex_ = 505 nm; λ_em_ = 650 nm.

For hematoxylin-eosin staining, the tumor samples and organs were collected and fixed with 4% paraformaldehyde at 4 °C. All the samples then were transferred to a 10% formalin-neutral buffer solution and embedded in paraffin. The sections were stained by hematoxylin-eosin (Beyotime, China) and observed under a Zeiss inverted fluorescence microscope, Germany.

All the optimization calculation was treated at B3LYP/6-31G(d,p) level and conducted in the Gaussian 09 package.^[4-6]^

1. Hariharan, P. C.; Pople, J. A. Accuracy of AH Nequilibrium Geometries by Single Determinant Molecular Orbital Theory. Molecular Physics 1974, 27 (1), 209–214.
2. Petersson, G. A.; Bennett, A.; Tensfeldt, T. G.; Laham, Al, M. A.; Shirley, W. A.; Mantzaris, J. A Complete Basis Set Model Chemistry. I. the Total Energies of Closed‐Shell Atoms and Hydrides of the First‐Row Elements. J. Chem. Phys. 1988, 89 (4), 2193–2218.
3. Gaussian 09, Revision A.02, M. J. Frisch, G. W. Trucks, H. B. Schlegel, G. E. Scuseria, M. A. Robb, J. R. Cheeseman, G. Scalmani, V. Barone, G. A. Petersson, H. Nakatsuji, X. Li, M. Caricato, A. Marenich, J. Bloino, B. G. Janesko, R. Gomperts, B. Mennucci, H. P. Hratchian, J. V. Ortiz, A. F. Izmaylov, J. L. Sonnenberg, D. Williams-Young, F. Ding, F. Lipparini, F. Egidi, J. Goings, B. Peng, A. Petrone, T. Henderson, D. Ranasinghe, V. G. Zakrzewski, J. Gao, N. Rega, G. Zheng, W. Liang, M. Hada, M. Ehara, K. Toyota, R. Fukuda, J. Hasegawa, M. Ishida, T. Nakajima, Y. Honda, O. Kitao, H. Nakai, T. Vreven, K. Throssell, J. A. Montgomery, Jr., J. E. Peralta, F. Ogliaro, M. Bearpark, J. J. Heyd, E. Brothers, K. N. Kudin, V. N. Staroverov, T. Keith, R. Kobayashi, J. Normand, K. Raghavachari, A. Rendell, J. C. Burant, S. S. Iyengar, J. Tomasi, M. Cossi, J. M. Millam, M. Klene, C. Adamo, R. Cammi, J. W. Ochterski, R. L. Martin, K. Morokuma, O. Farkas, J. B. Foresman, and D. J. Fox, Gaussian, Inc., Wallingford CT, 2016.

# 2. Supplementary figures

Scheme S1. Synthetic route of TQ-H_2_.


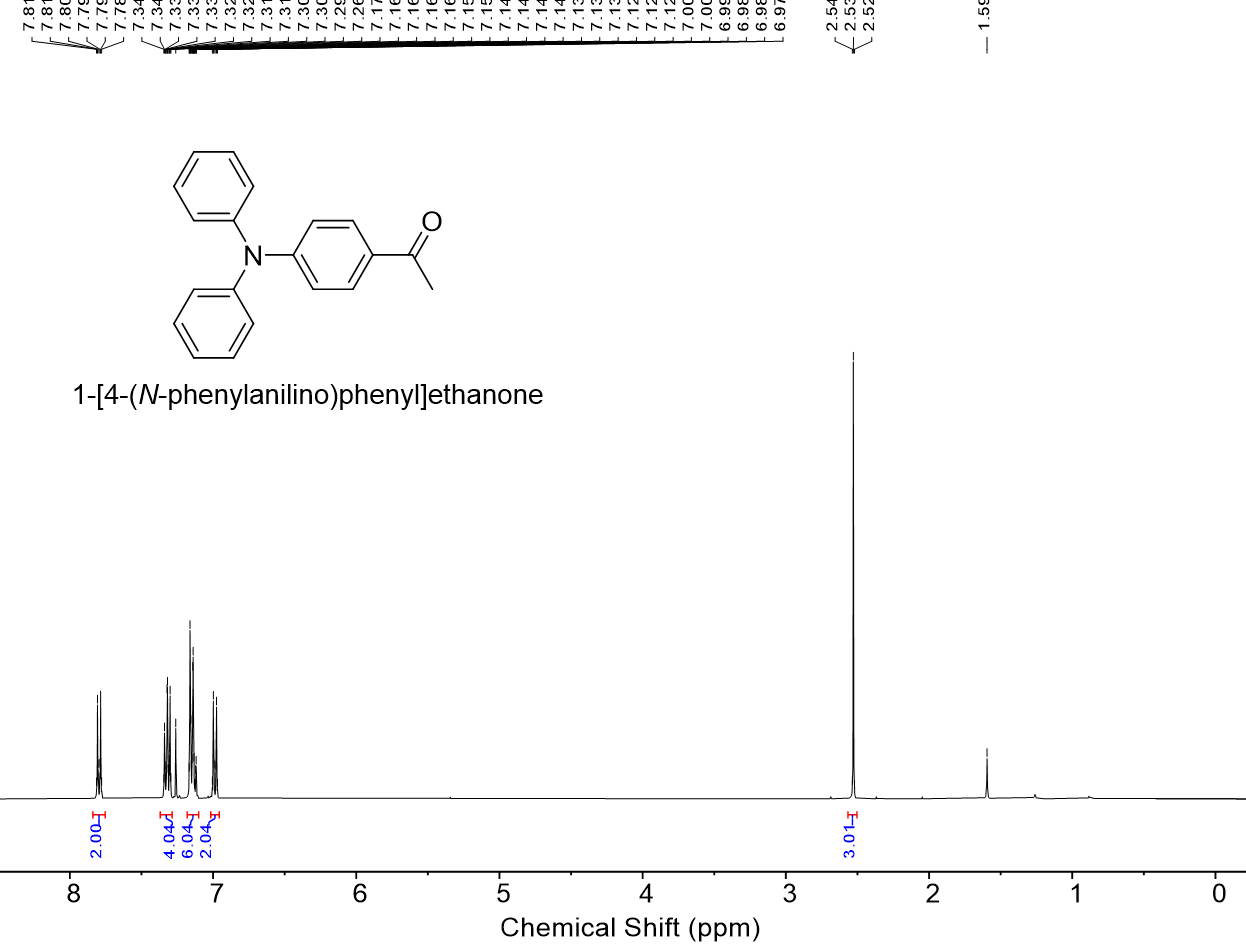


Figure S1. ^1^H NMR spectrum of 1-[4-(*N*-phenylanilino)phenyl]ethenone in CDCl_3_.


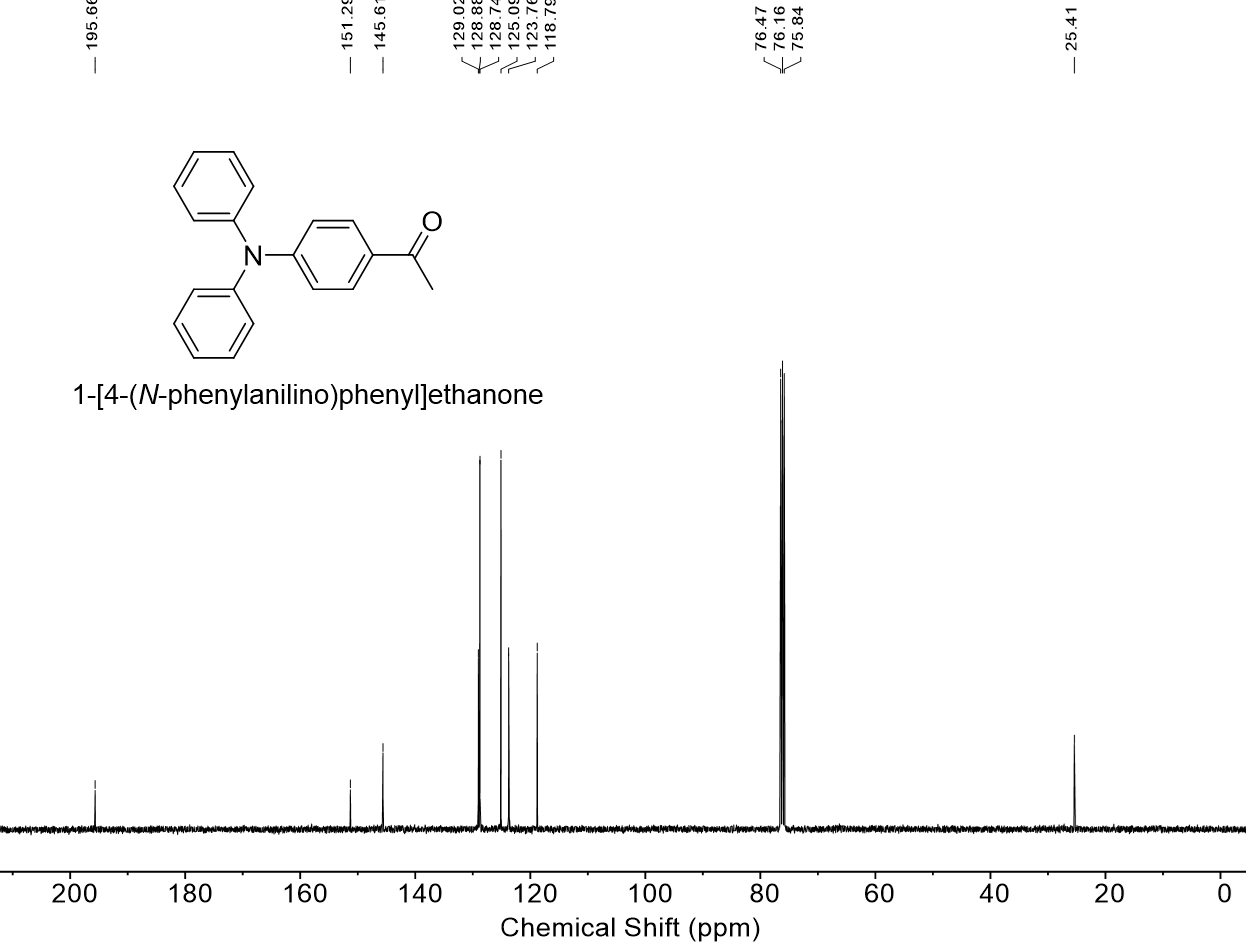


Figure S2. ^13^C NMR spectrum of 1-(4-(diphenylamino)phenyl)ethan-1-one in CDCl_3_.


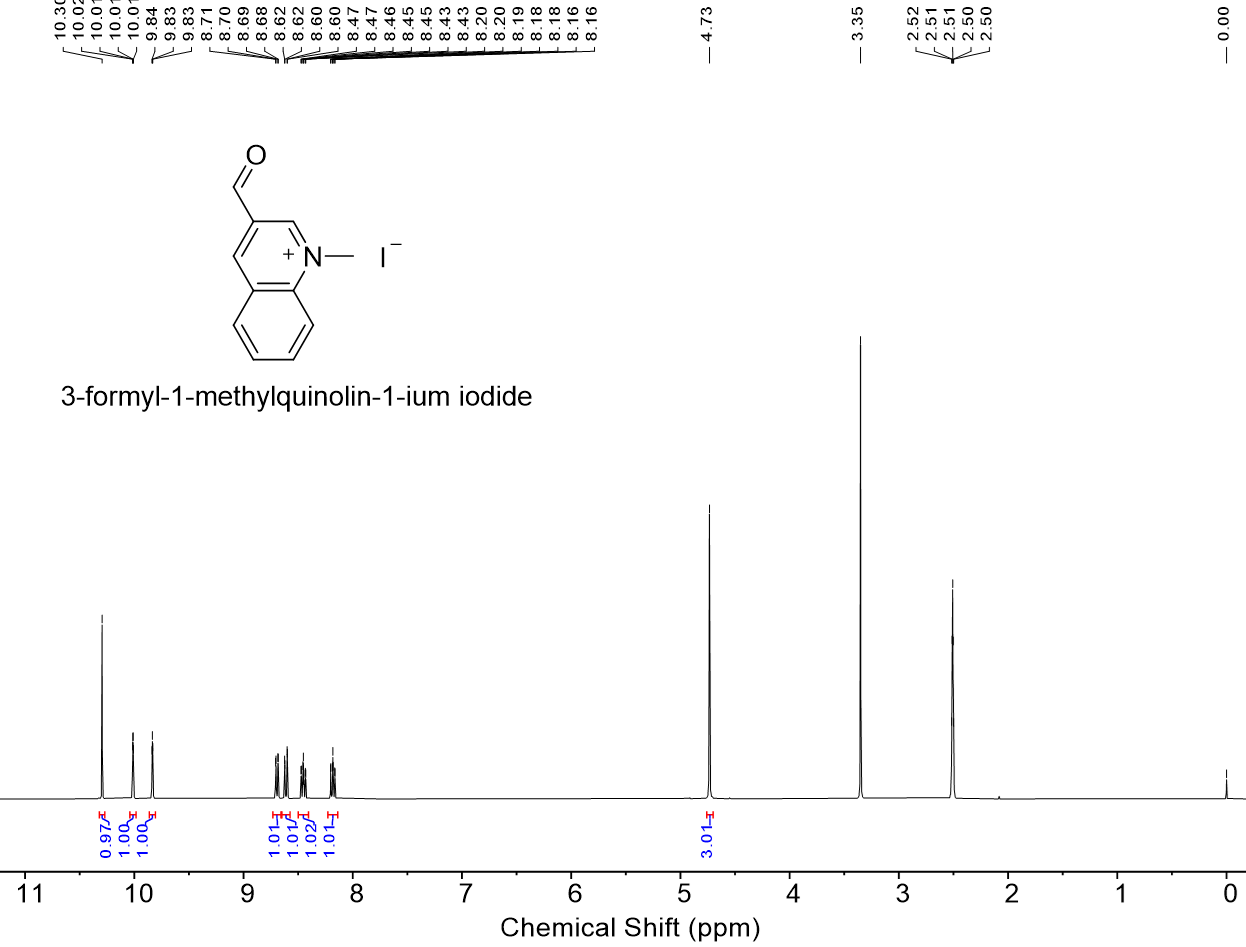


Figure S3. ^1^H NMR spectrum of 3-formyl-1-methylquinolin-1-ium iodide in CDCl_3_.


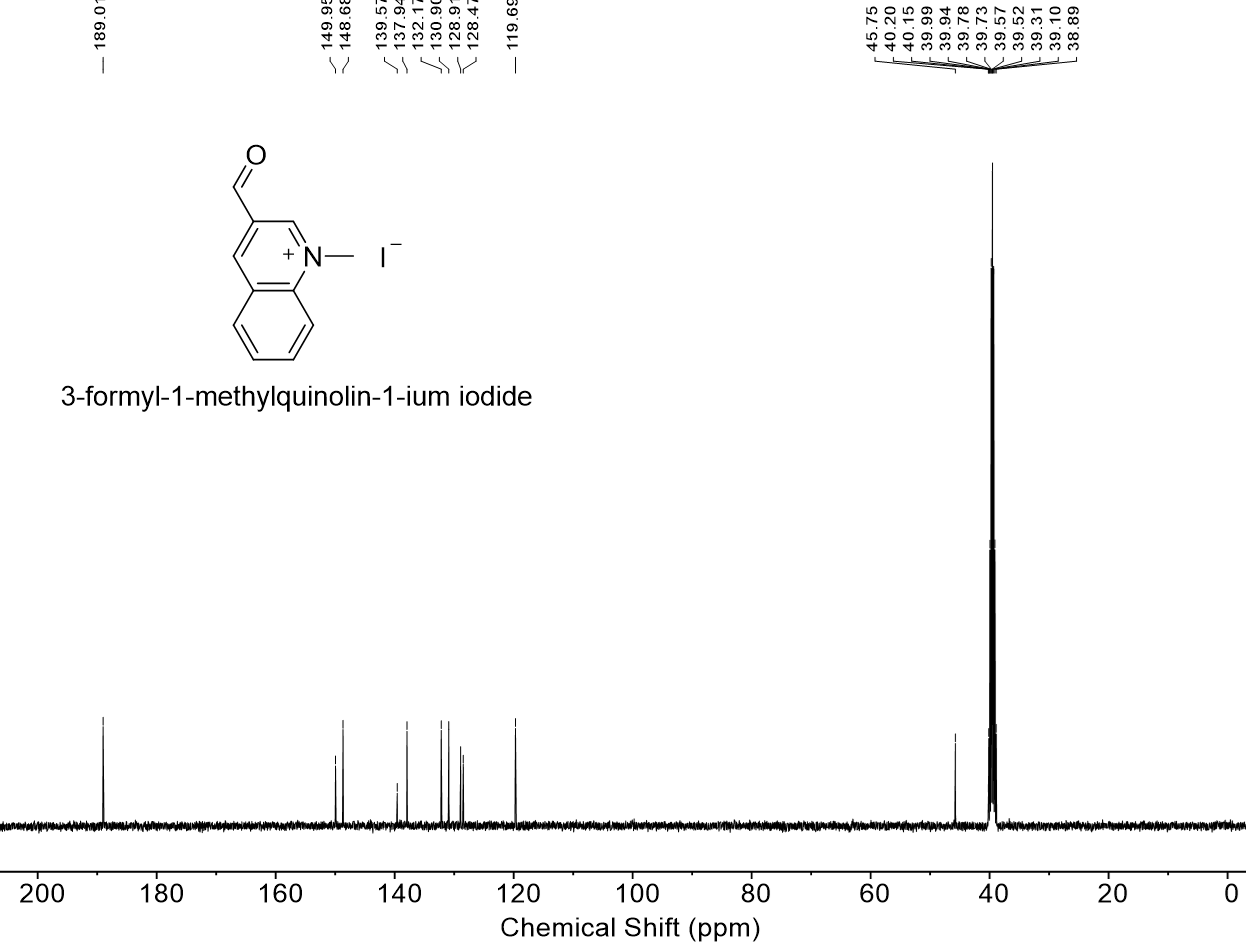


Figure S4. ^13^C NMR spectrum of 3-formyl-1-methylquinolin-1-ium iodide in CDCl_3_.


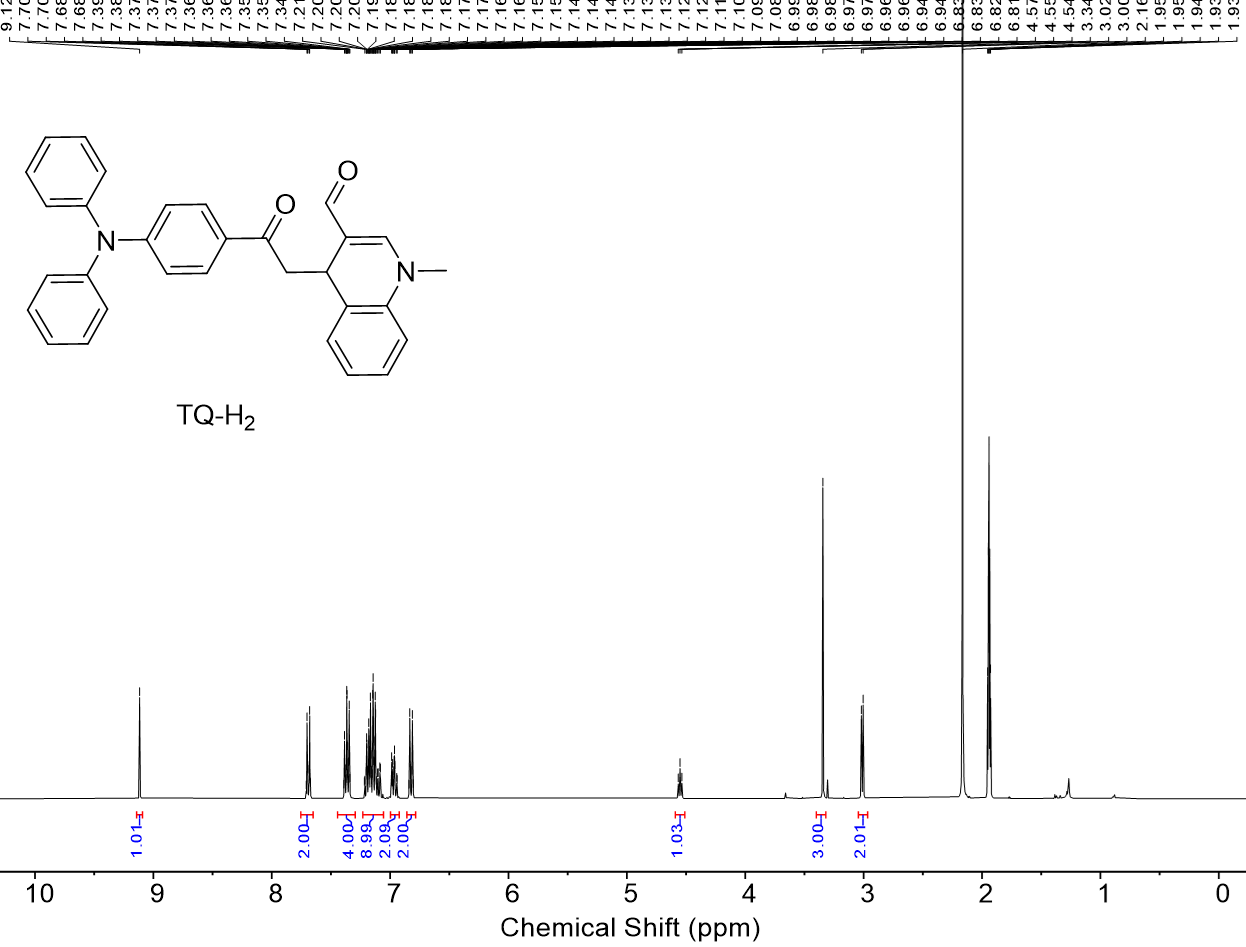


Figure S5. ^1^H NMR spectrum of TQ-H_2_ in CD_3_CN.


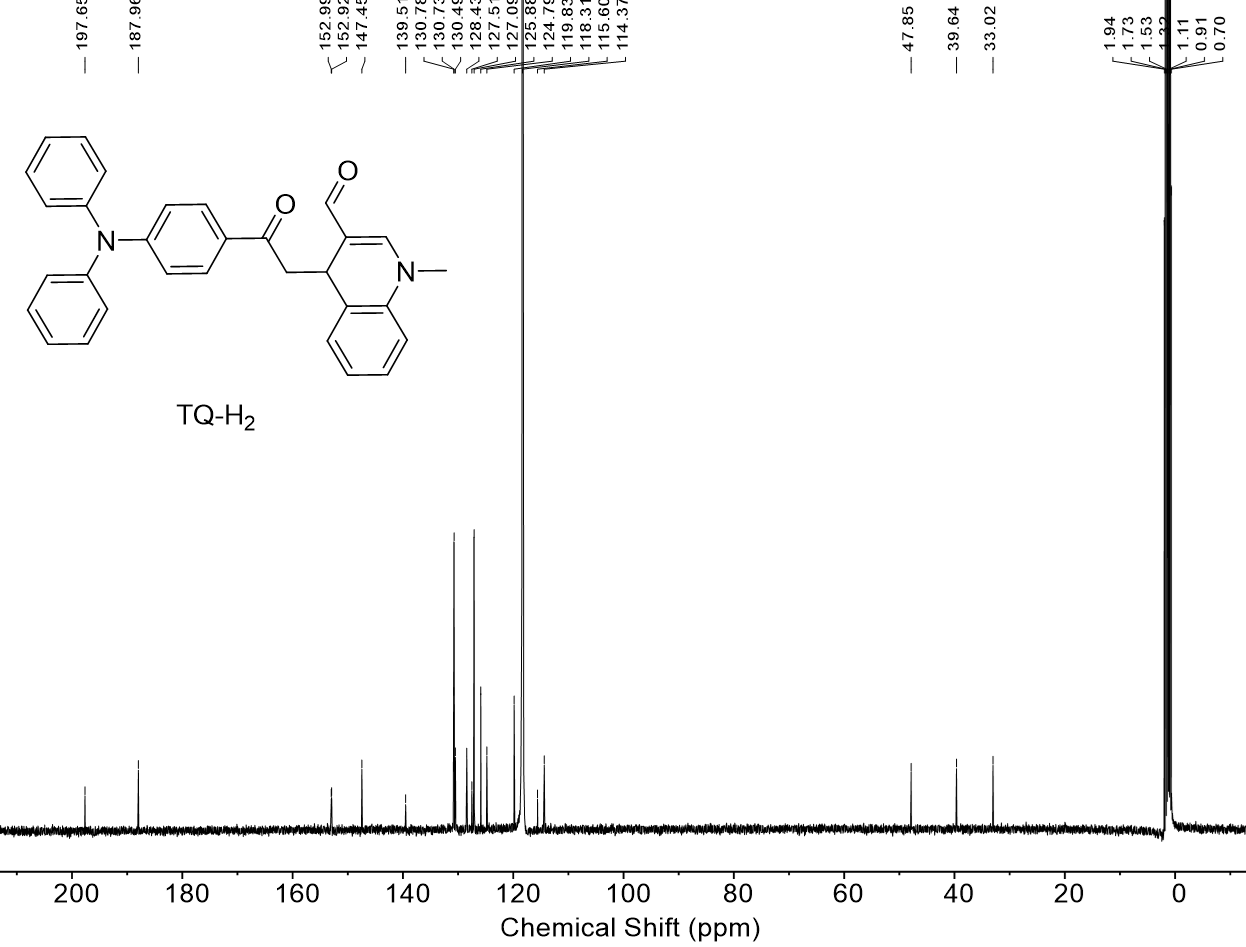


Figure S6. ^13^C NMR spectrum of TQ-H_2_ in CD_3_CN.


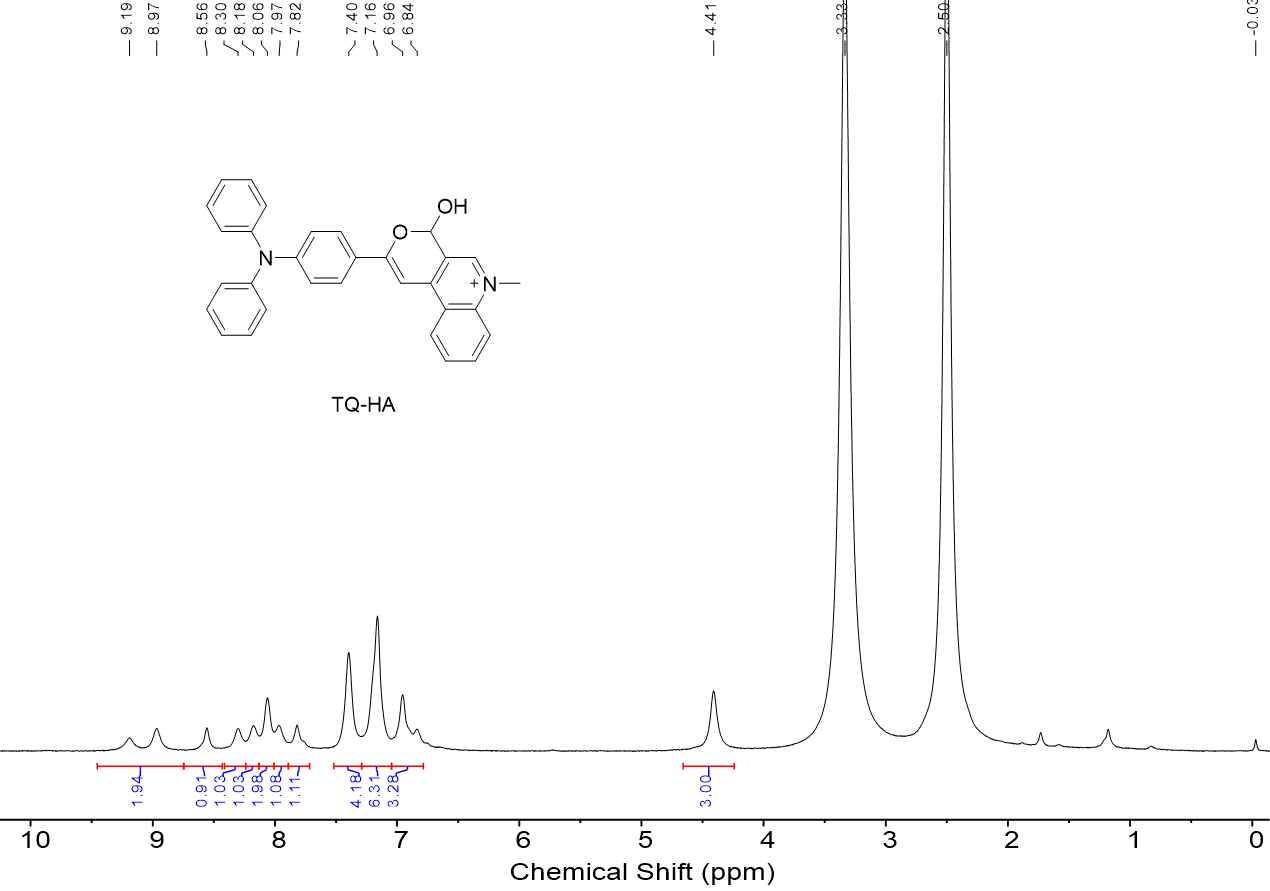


Figure S7. ^1^H NMR spectrum of TQ-HA in DMSO-*d*_6_.


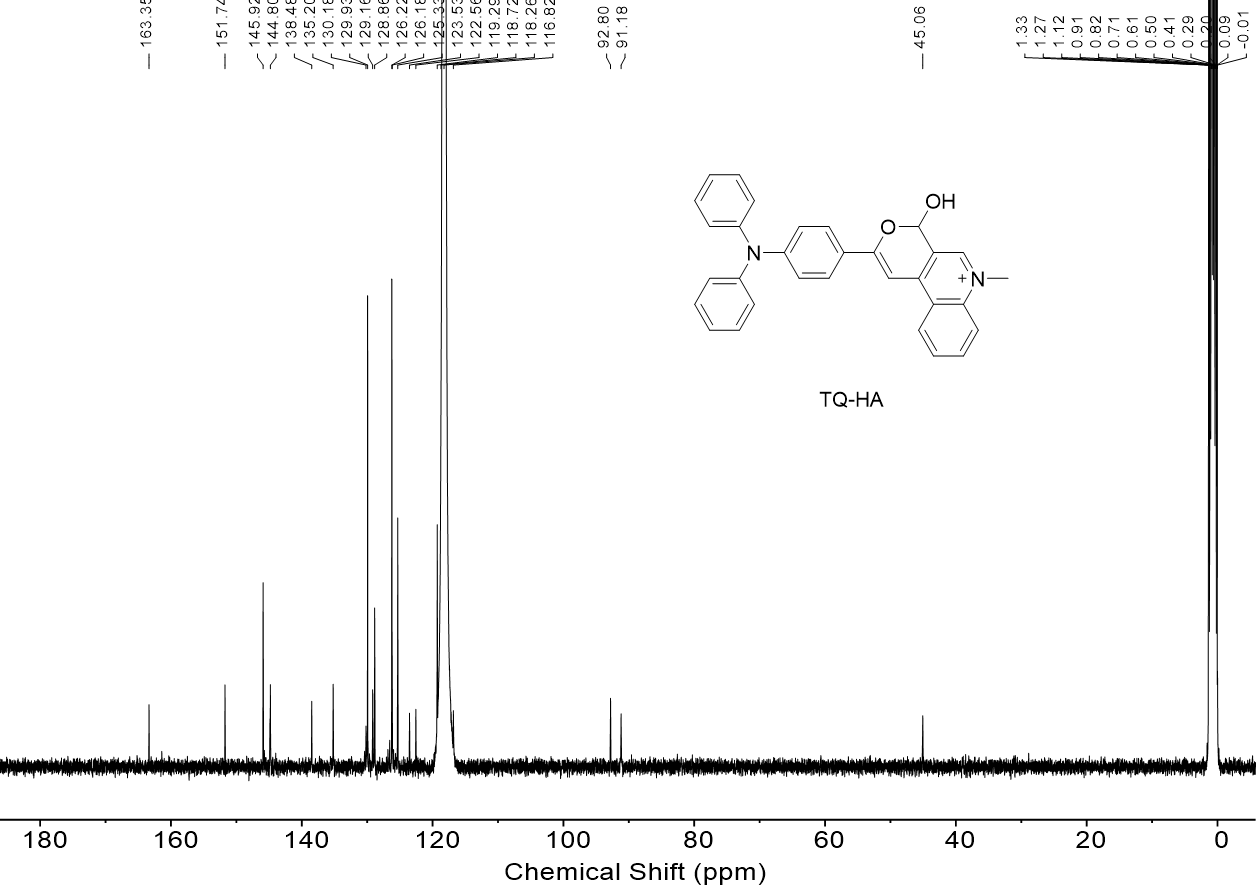


Figure S8. ^13^C NMR spectrum of TQ-HA in CD_3_CN/D_2_O.


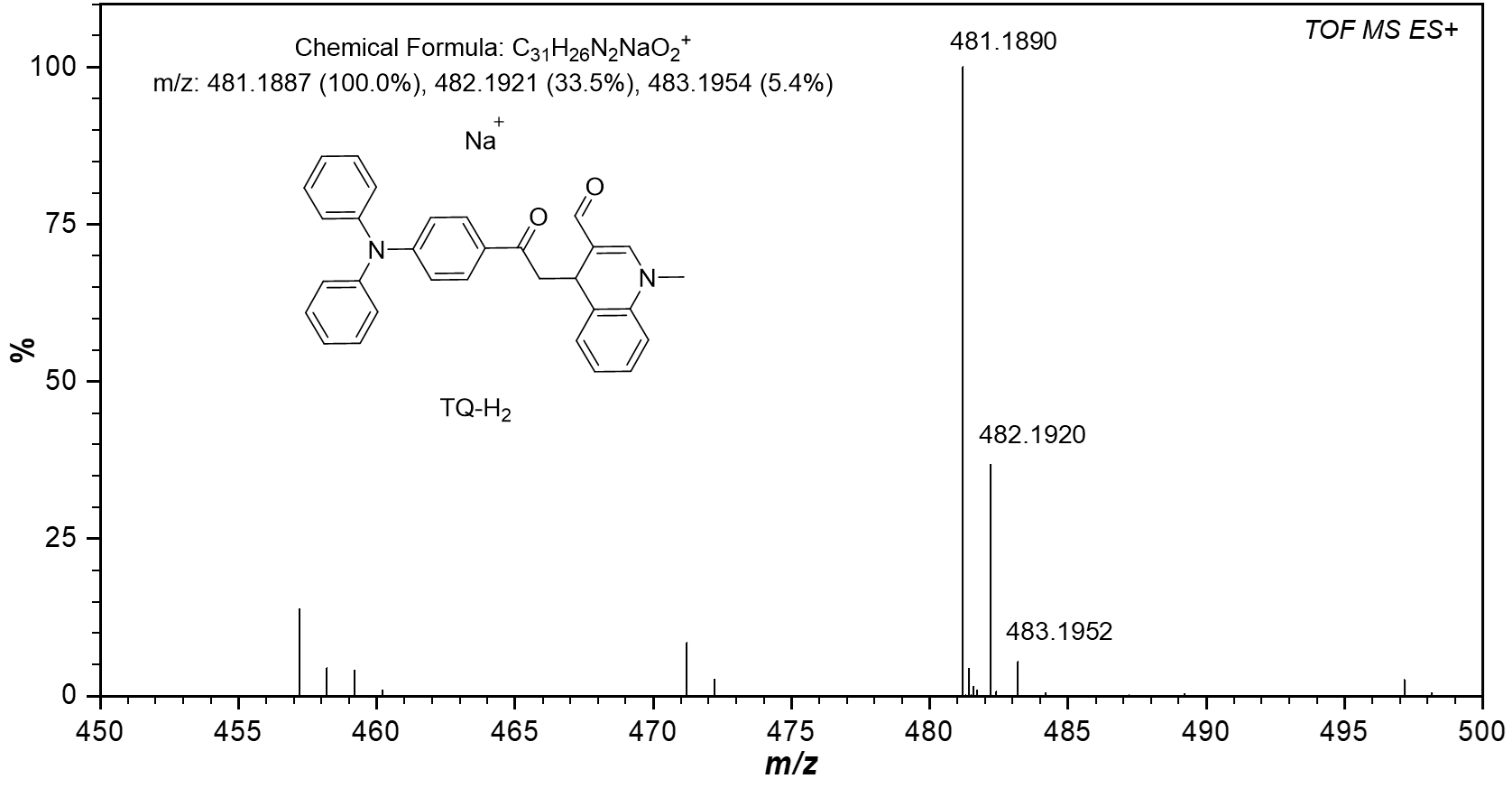
Figure S9. HRMS spectrum of TQ-H_2_.


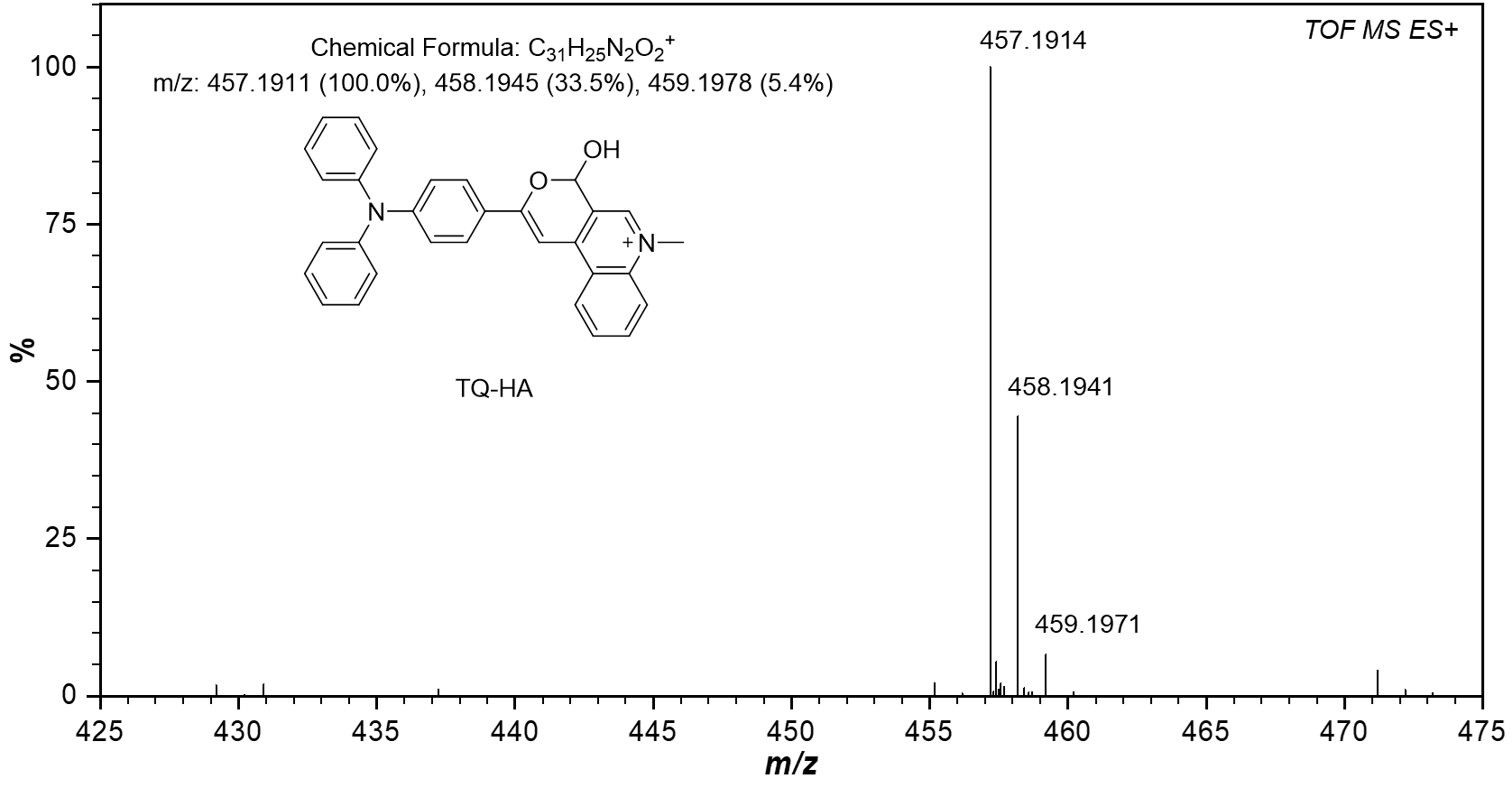
Figure S10. HRMS spectrum of TQ-HA.


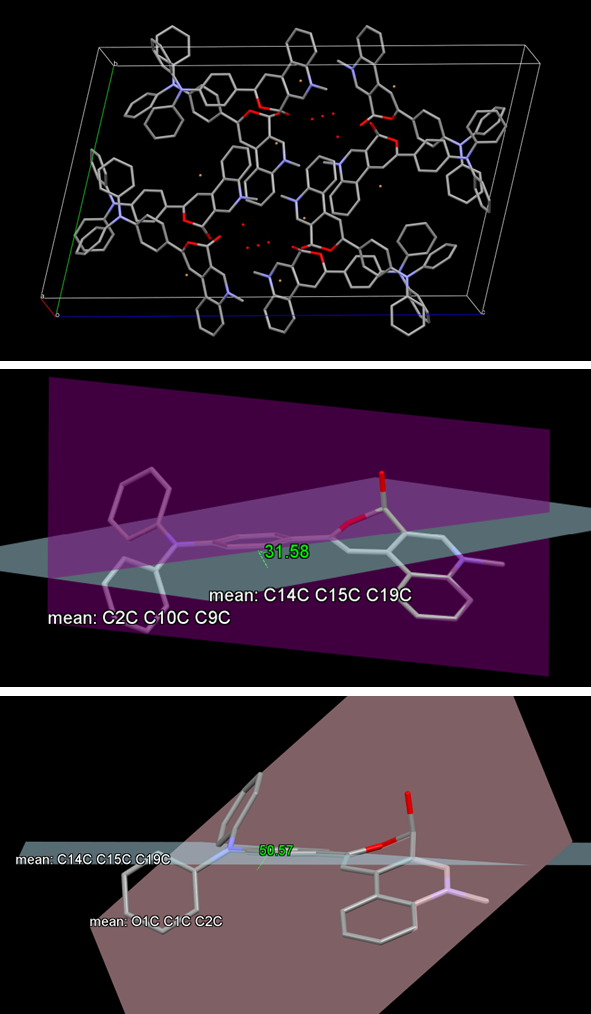


Figure S11. Single crystal structure of TQ-HA.


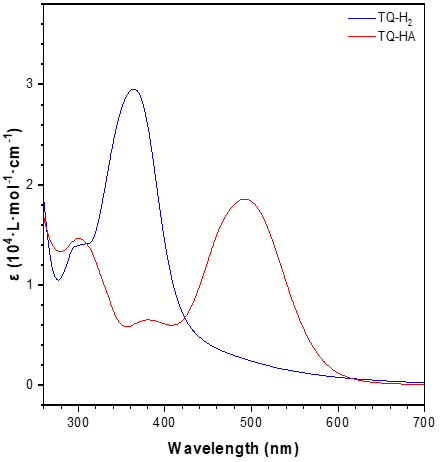


Figure S12. Absorption spectra of TQ-H_2_ and TQ-HA in 7.0 PBS.


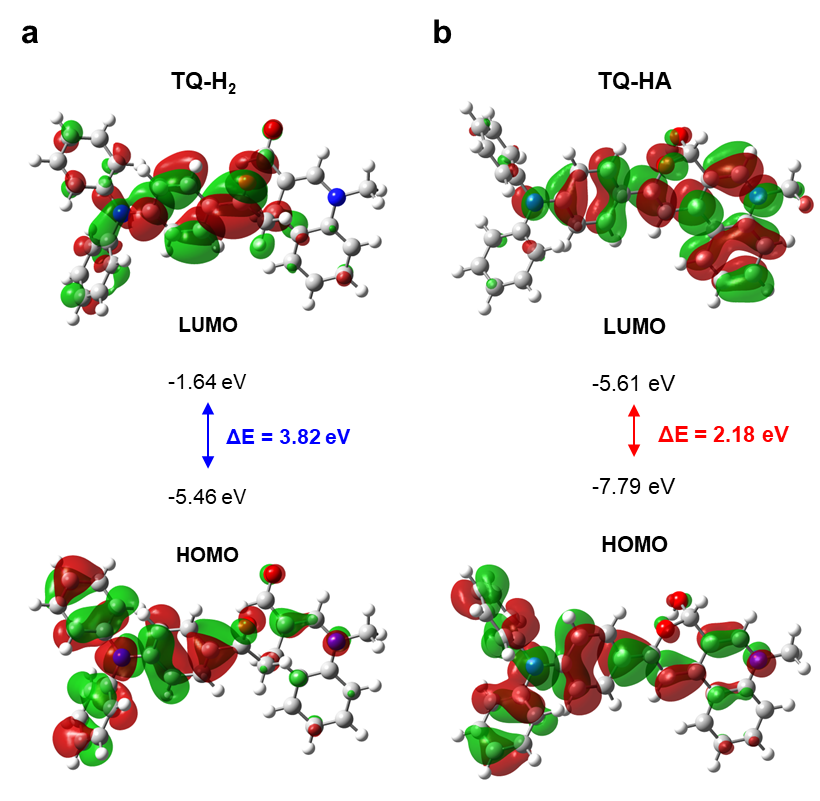


Figure S13. HOMO and LUMO distribution of (a) TQ-H_2_ and (b) TQ-HA calculated by DFT.


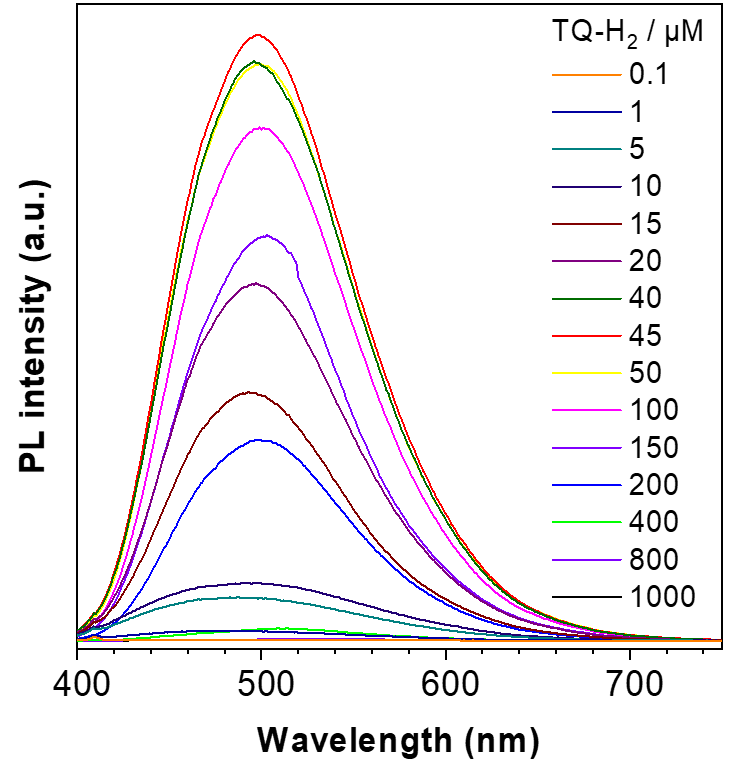


Figure S14. PL spectra of TQ-H_2_ in acetone with different concentrations. Excitation wavelength = 365 nm.


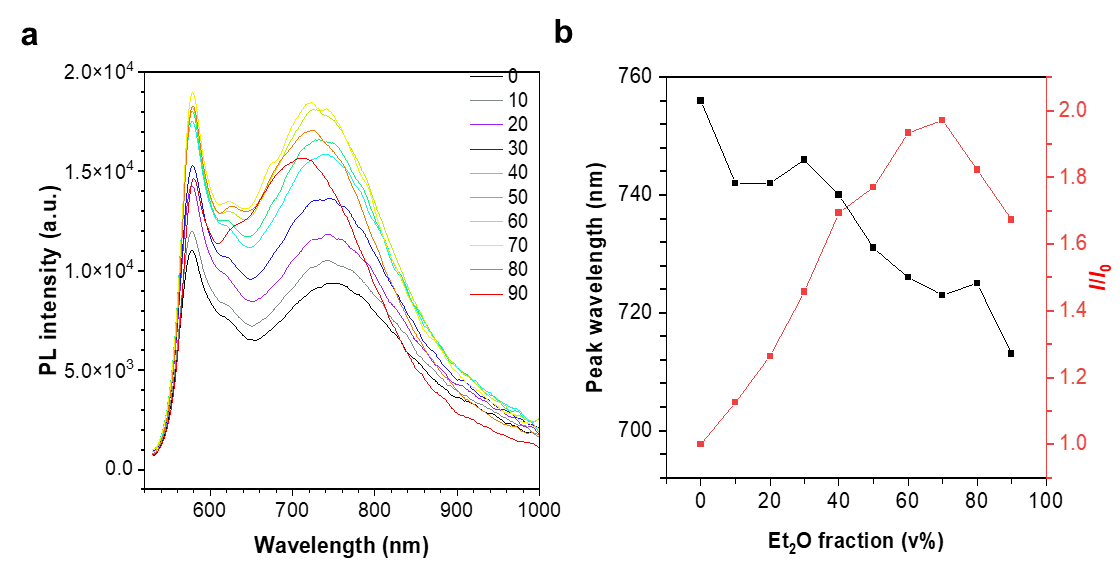


Figure S15. (a) PL spectra of TQ-HA (10 μM) in ACN/diethyl ether mixtures with different ether fractions (*f*_Etrher_). (b) Change in relative PL peak intensity (*I*/*I*_0_) and wavelength of TQ-HA in ACN/diethyl ether mixtures with different *f*_Etrher_, where *I*_0_ was the PL peak intensity in ACN solution.


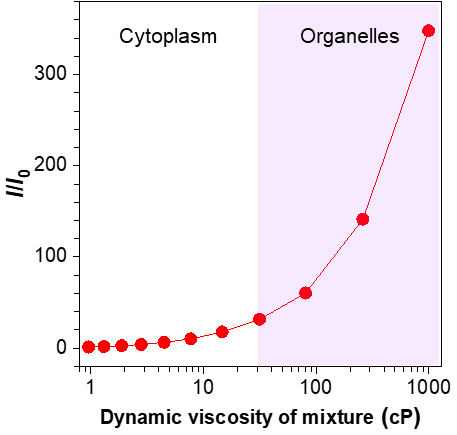


Figure S16. PL spectra of TQ-HA (10 μM) in water/glycerol mixtures with different viscosity. Excitation wavelength: 510 nm.

Table S1. The photophysical properties of TQ-H_2_ and TQ-HA


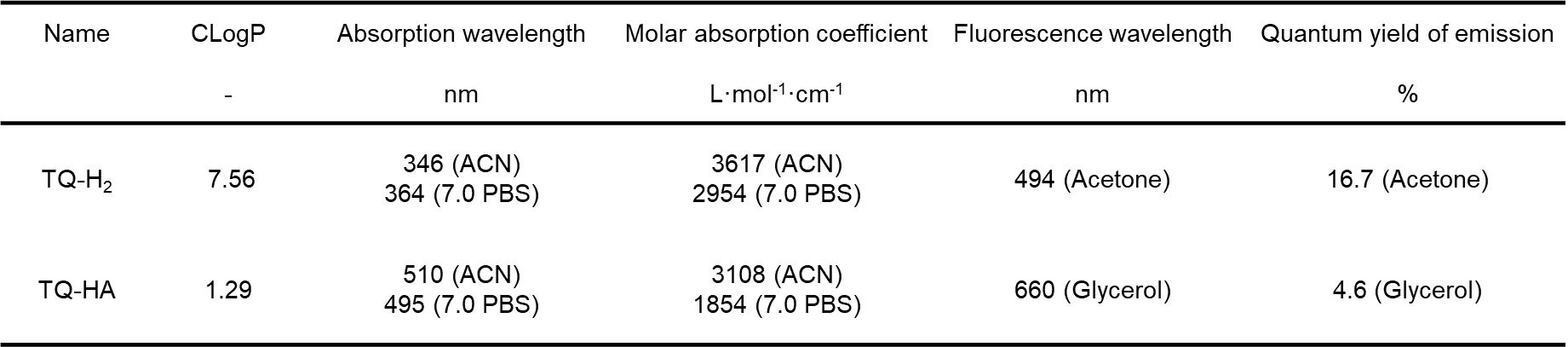


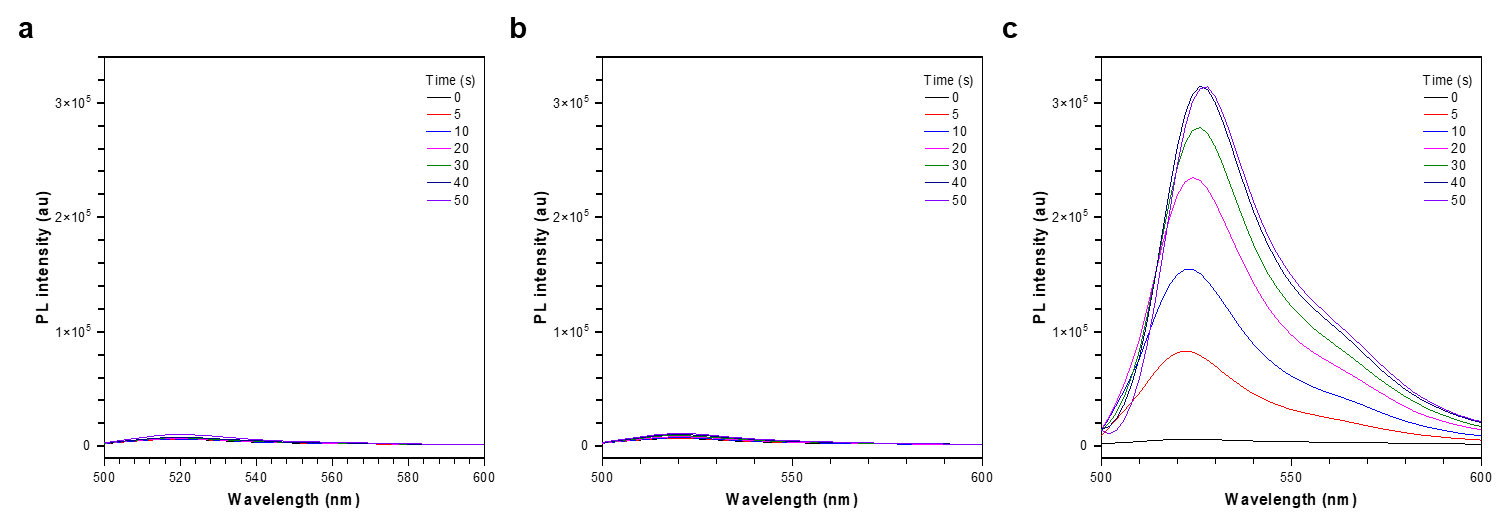


Figure S17. PL spectra of DCFH in the (a) absence and presence of (b) TQ-H_2_, and (c) TQ-HA in pH 7.0 PBS solution (with 1 vol % DMSO) under light irradiation (5 mW/cm^2^). [TQ-H_2_] = 10 μM, [TQ-HA] = 10 μM, [DCFH] = 50 μM.


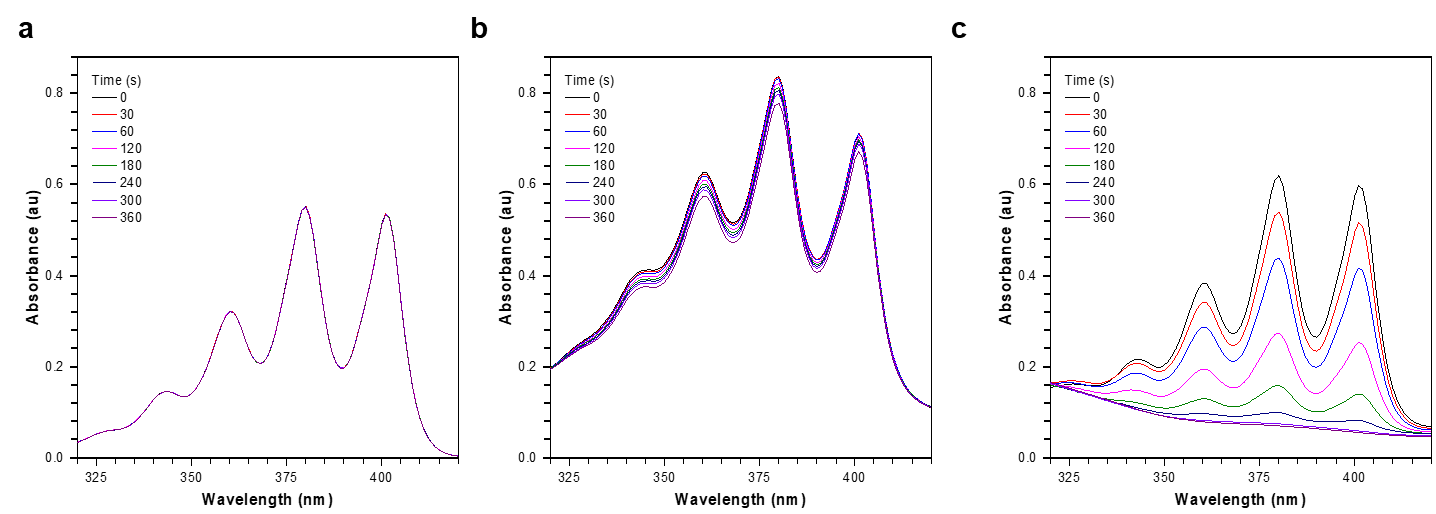


Figure S18. Absorption spectra of ABDA in the (a) absence and presence of (b) TQ-H_2_, and (c) TQ-HA in pH 7.0 PBS solution (with 1 vol % DMSO) under light irradiation (5 mW/cm^2^). [TQ-H_2_] = 10 μM, [TQ-HA] = 10 μM, [ABDA] = 50 μM.


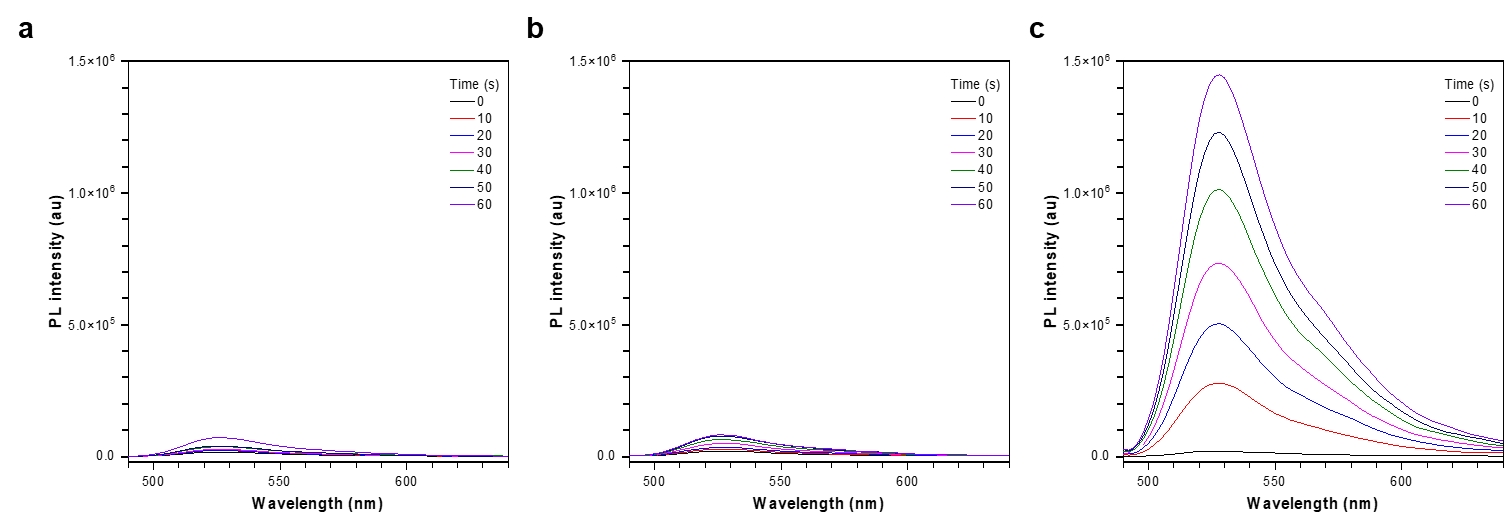


Figure S19. PL spectra of DHR123 in the (a) absence and presence of (b) TQ-H_2_, and (c) TQ-HA in pH 7.0 PBS solution (with 1 vol % DMSO) under light irradiation (5 mW/cm^2^). [TQ-H_2_] = 10 μM, [TQ-HA] = 10 μM, [DHR123] = 20 μM.


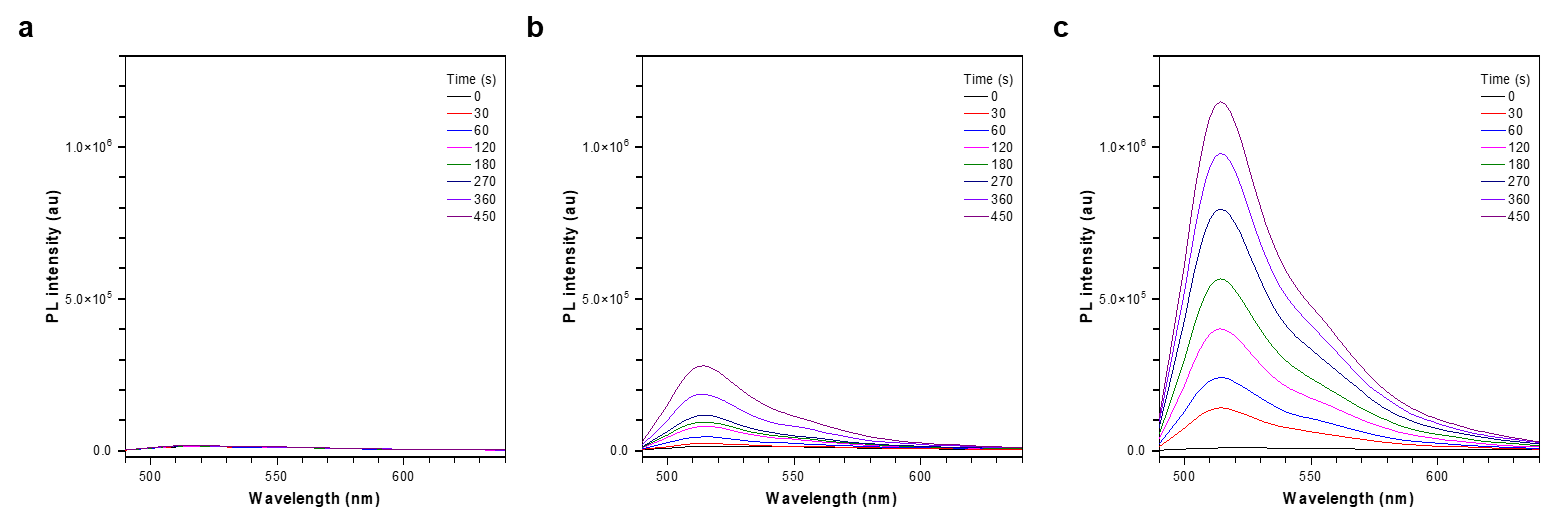


Figure S20. PL spectra of HPF in the (a) absence and presence of (b)TQ-H_2_, and (c) TQ-HA in pH 7.0 PBS solution (with 1 vol % DMSO) under light irradiation (5 mW/cm^2^). [TQ-H_2_] = 10 μM, [TQ-HA] = 10 μM, [HPF] = 10 μM.


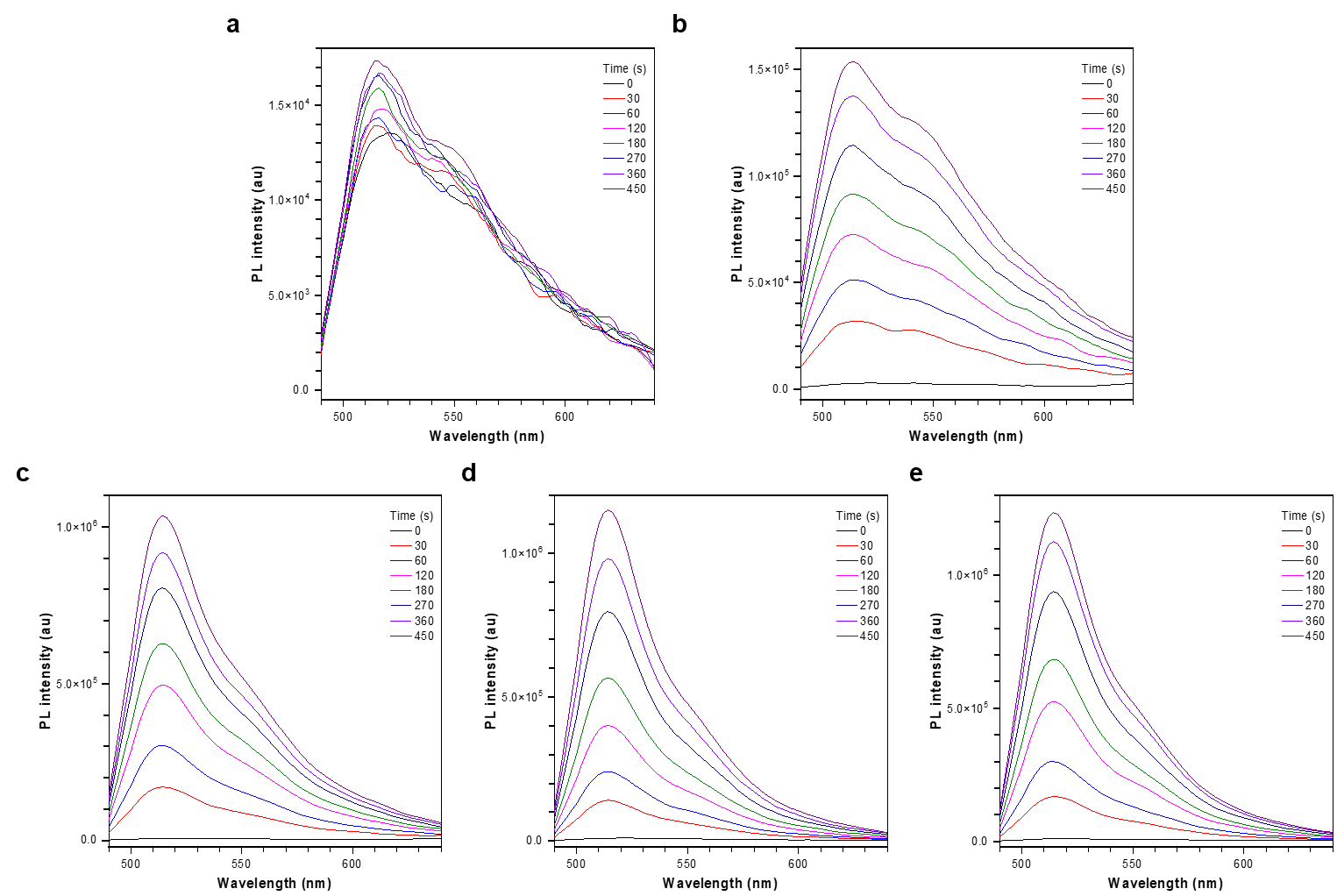


Figure S21. PL spectra of HPF in the (a) absence of TQ-HA in 7.0 PBS solution (with 1 vol % DMSO) and presence of TQ-HA in pH (b) 5.0, (c) 6.0, (d) 7.0, and (e) 7.4 PBS solution (with 1 vol % DMSO) under light irradiation (5 mW/cm^2^). [TQ-HA] = 10 μM, [HPF] = 10 μM.


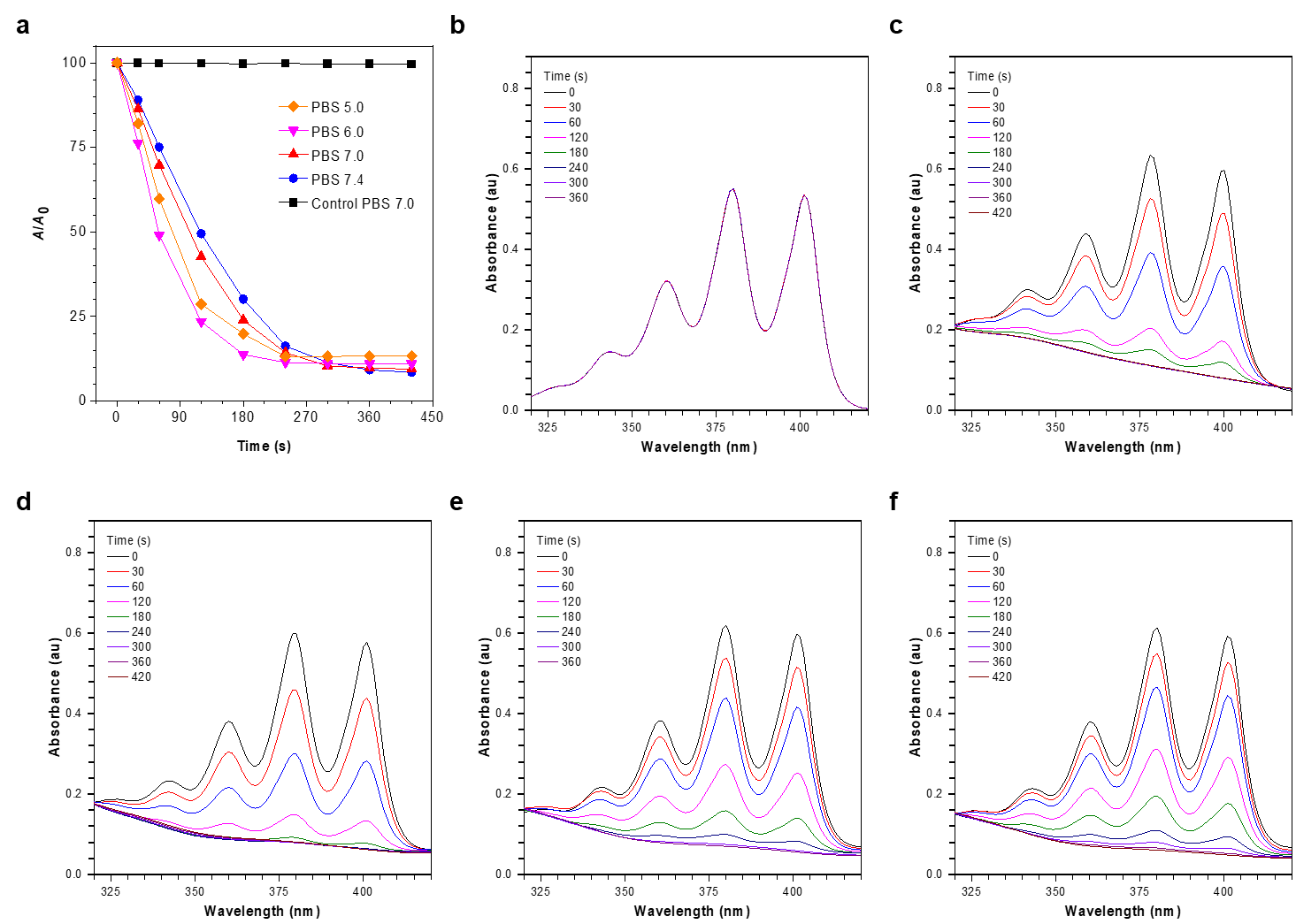


Figure S22. Time-course plots of ABDA decomposition in the presence of TQ-HA in different pH PBS solutions. Absorption spectra of ABDA in the (b) absence of TQ-HA in 7.0 PBS solution (with 1 vol % DMSO) and presence of TQ-HA in pH (c) 5.0, (d) 6.0, (e) 7.0, and (f) 7.4 PBS solution (with 1 vol % DMSO) under light irradiation (5 mW/cm^2^). [TQ-HA] = 10 μM, [ABDA] = 50 μM.


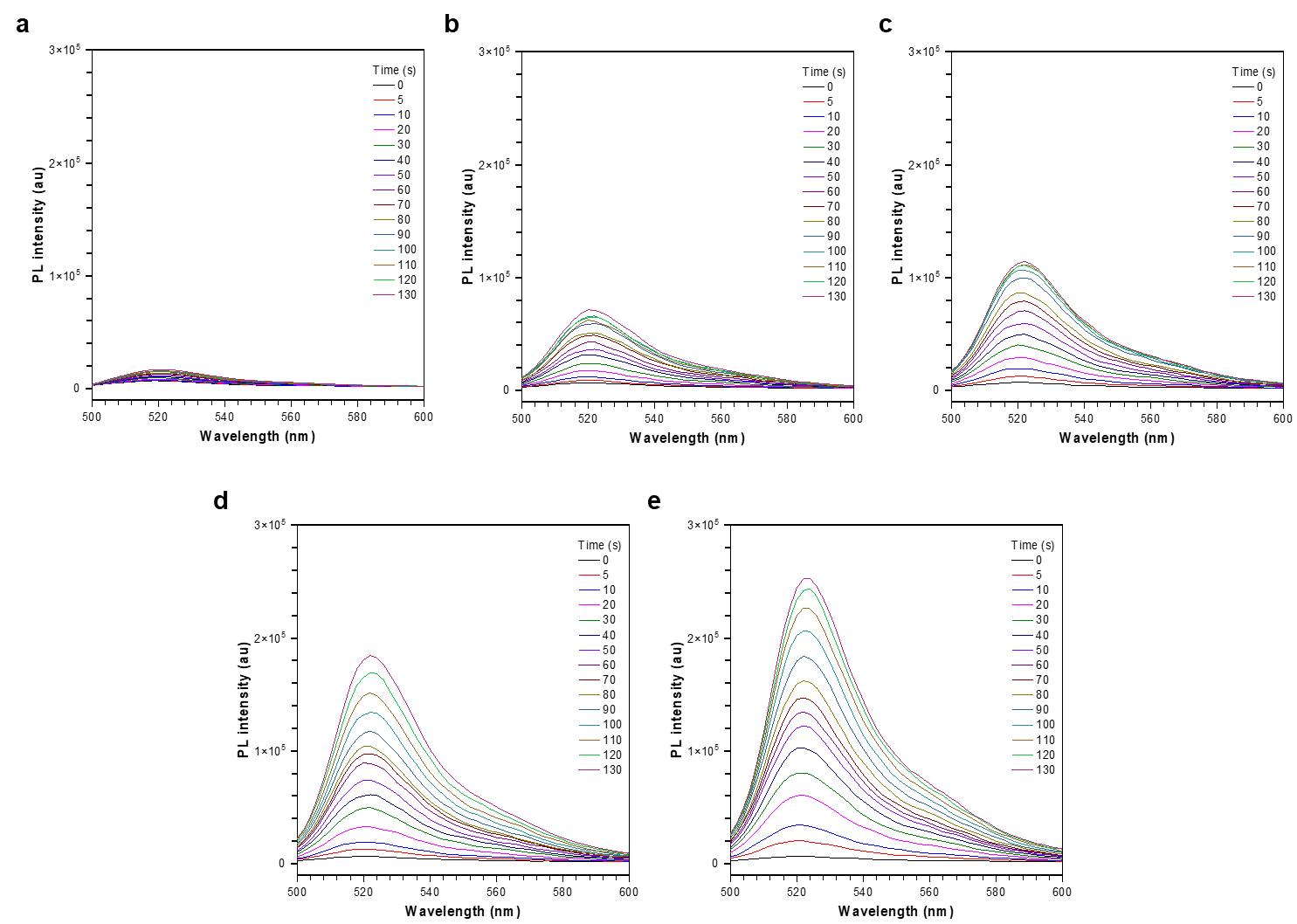


Figure S23. PL spectra of DCFH in the presence of (a) 10 μM TQ-H_2_, (b) 0.5 μM TQ-H_2_, (c) 0.5 μM TQ-H_2_ + 10 μM TQ-HA, (d) 1 μM TQ-H_2_, (e) 1 μM TQ-H_2_ + 10 μM TQ-HA in 7.0 PBS solution (with 1 vol % DMSO) under light irradiation (5 mW/cm^2^). [DCFH] = 50 μM.


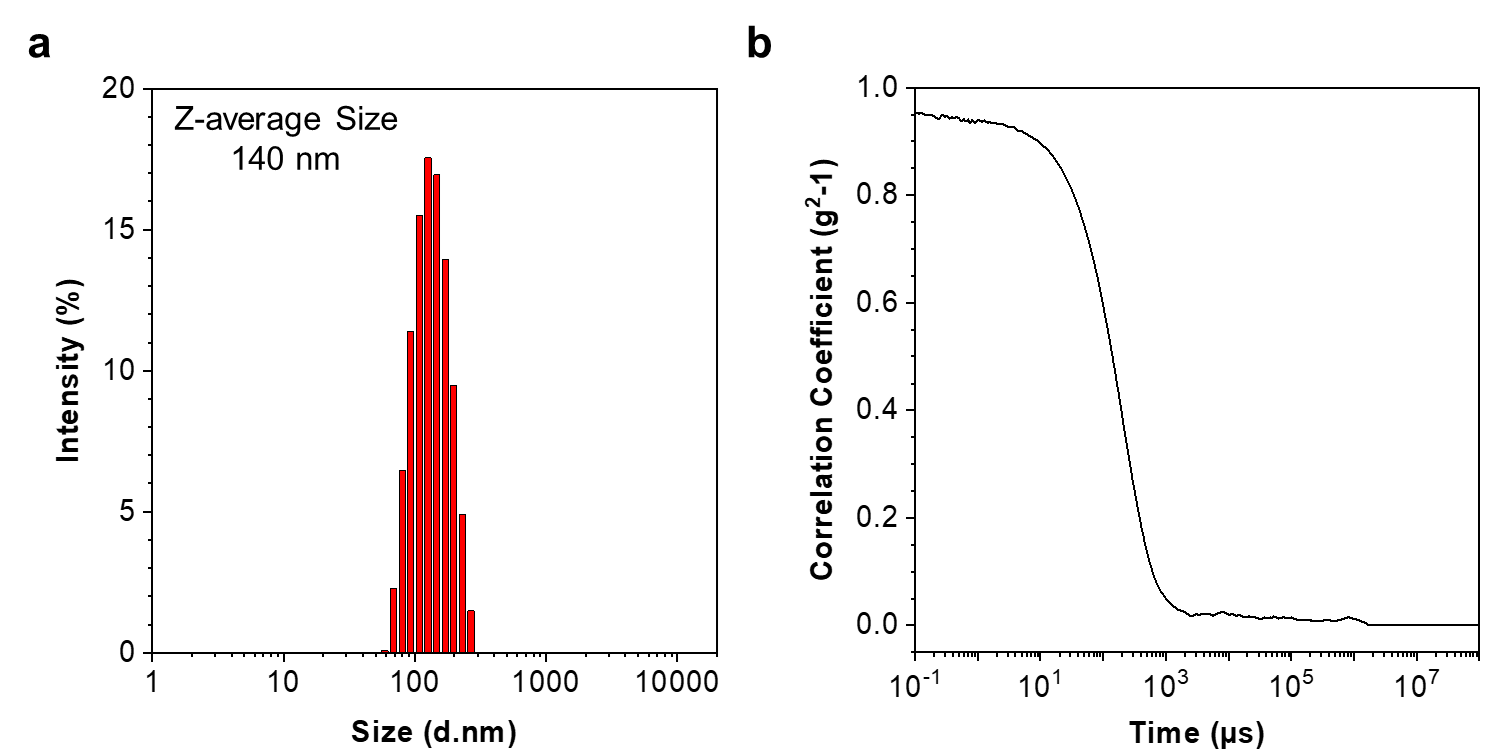


Figure S24. (a) Dynamic light scattering (DLS) result of TQ-H_2_ (10 μM) in DMSO/H_2_O mixture (1/100, v/v). (b) Variation of the intensity correlation function with time of DLS result of (a).


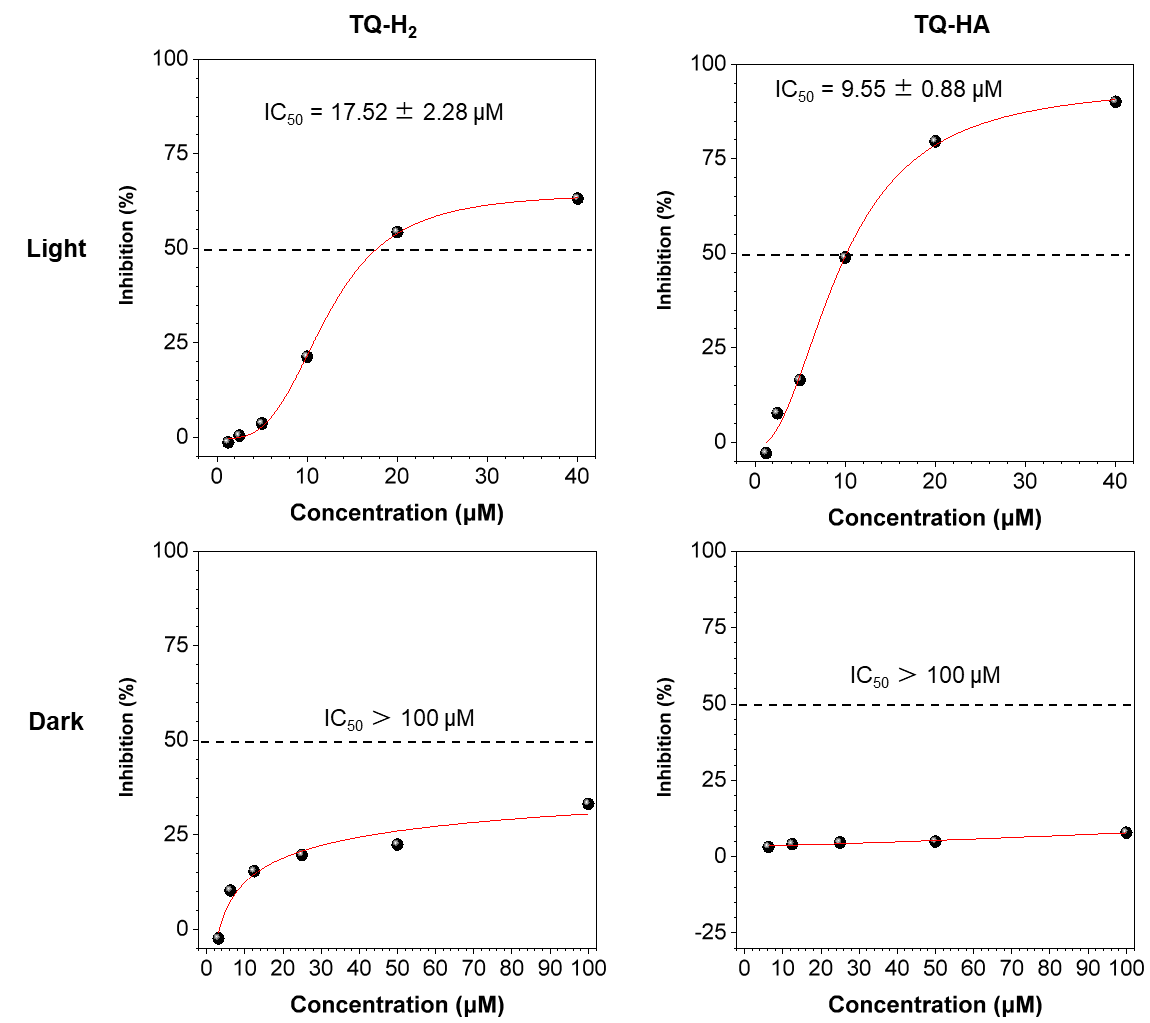


Figure S25. The IC50 tests of TQ-H_2_ and TQ-HA of LO2 cells with or without light irradiation.


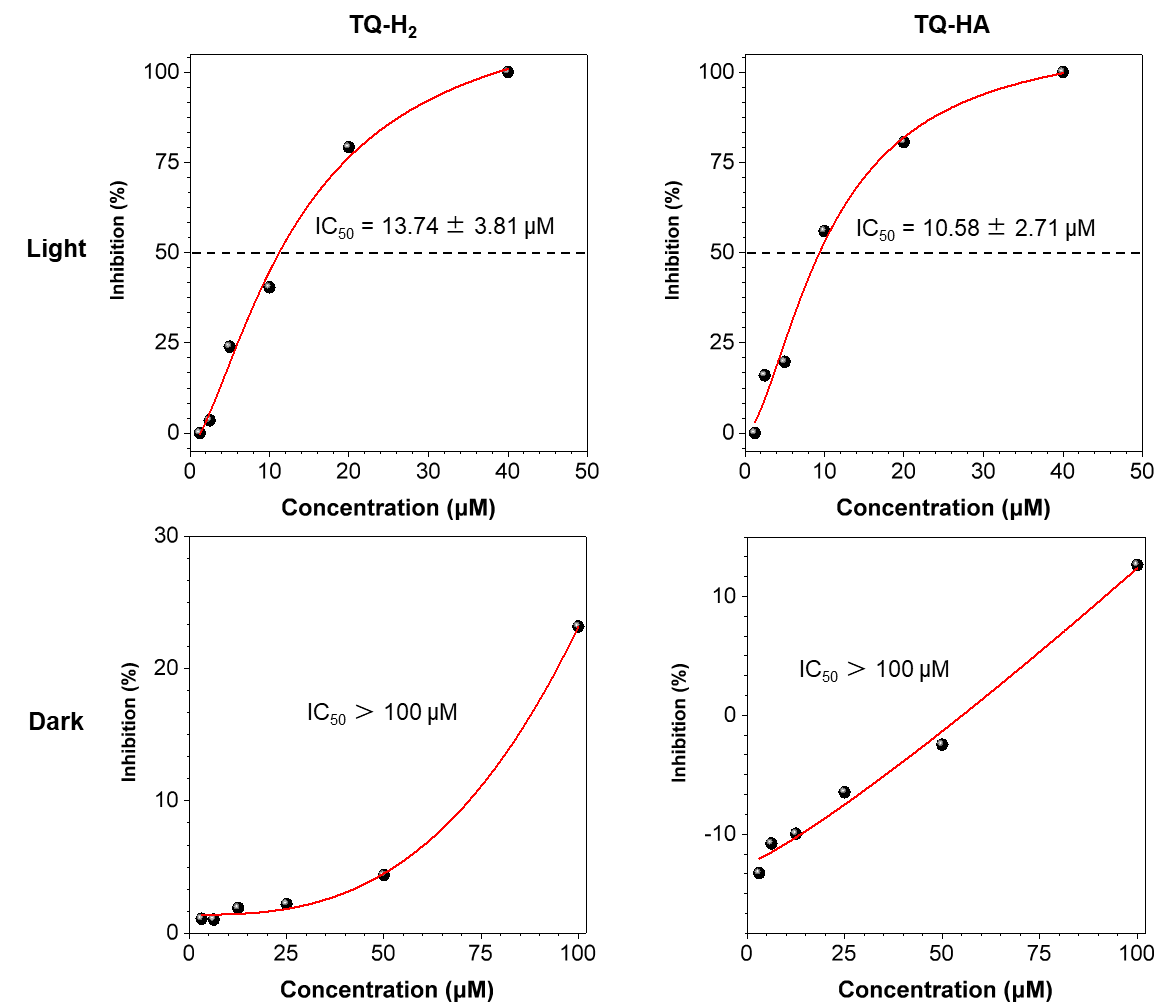


Figure S26. The IC50 tests of TQ-H_2_ and TQ-HA of HeLa cells with or without light irradiation.


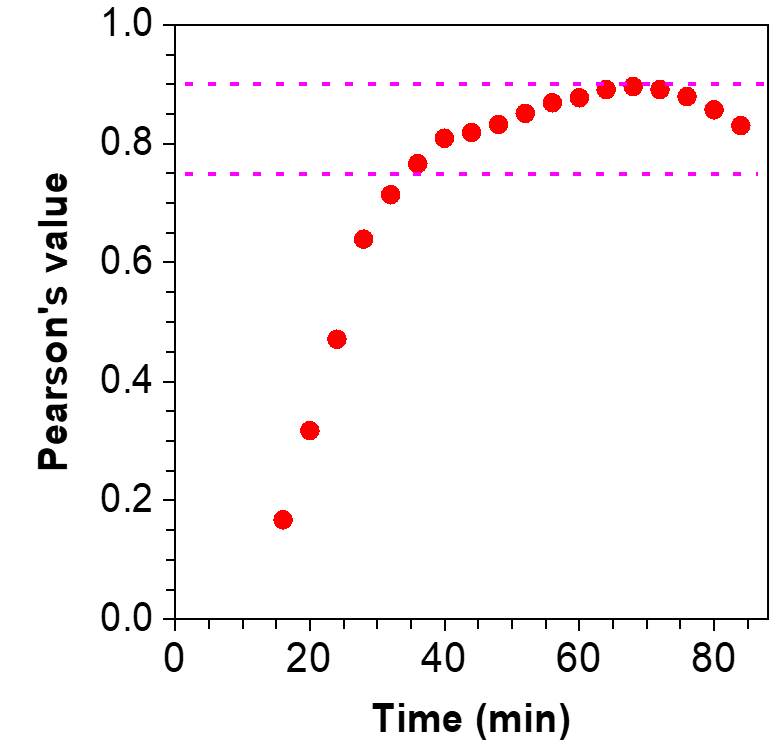


Figure S27. Quantitative analysis of colocalization using ZEISS ZEN and Image J.


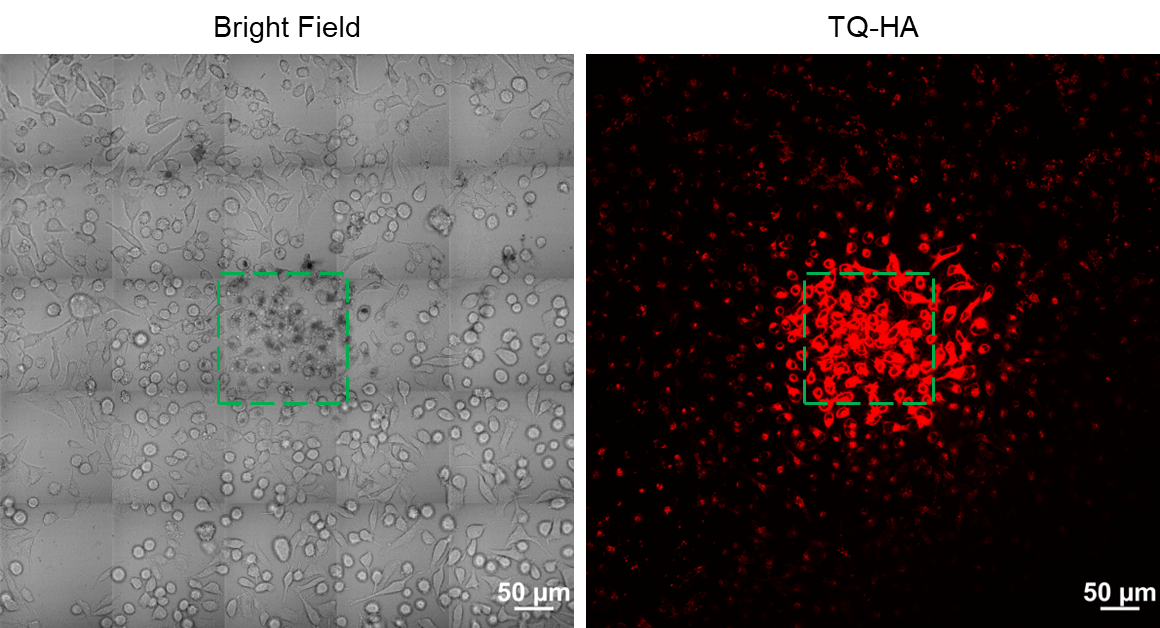


Figure S28. CLSM images of HeLa cells stained with TQ-H_2_ (5 μM) without washing and sequential scanning of the center area (green box) by using a 488 nm laser with 1% power for 1 h.


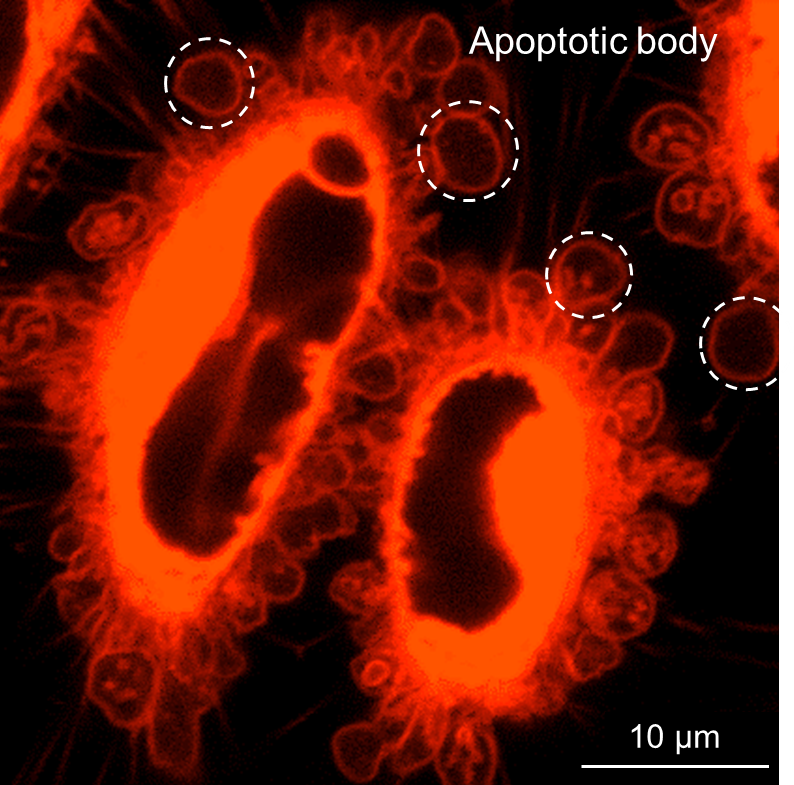


Figure S29. Local magnification of cells at the time of apoptosis.


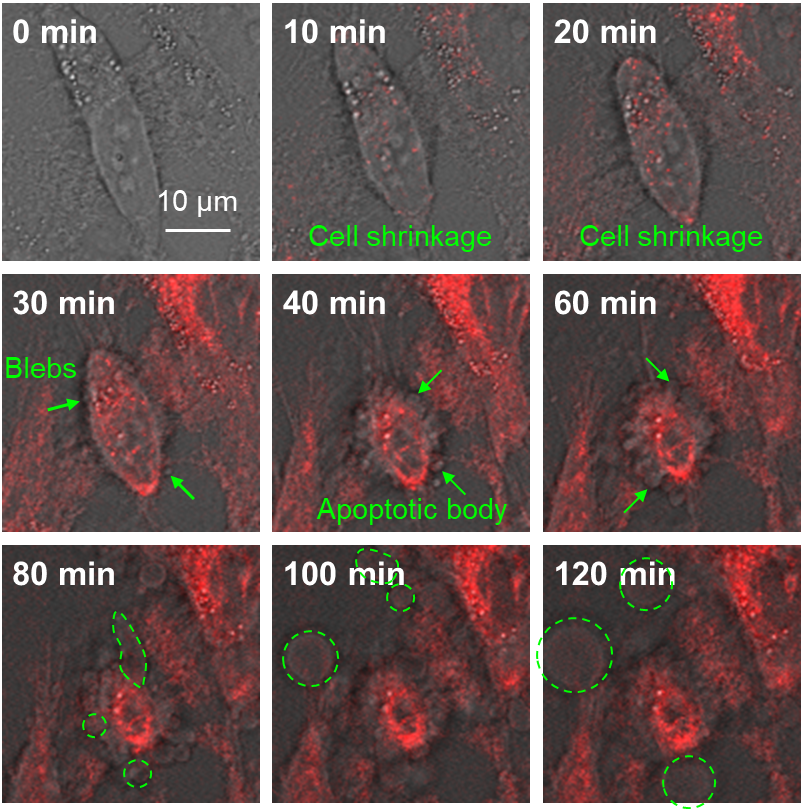


Figure S30. Real-time monitoring of HeLa cells stained with 5 μM TQ-H_2_ for 2 h without washing under continuous excitation by using a 405 nm laser with 2% power.


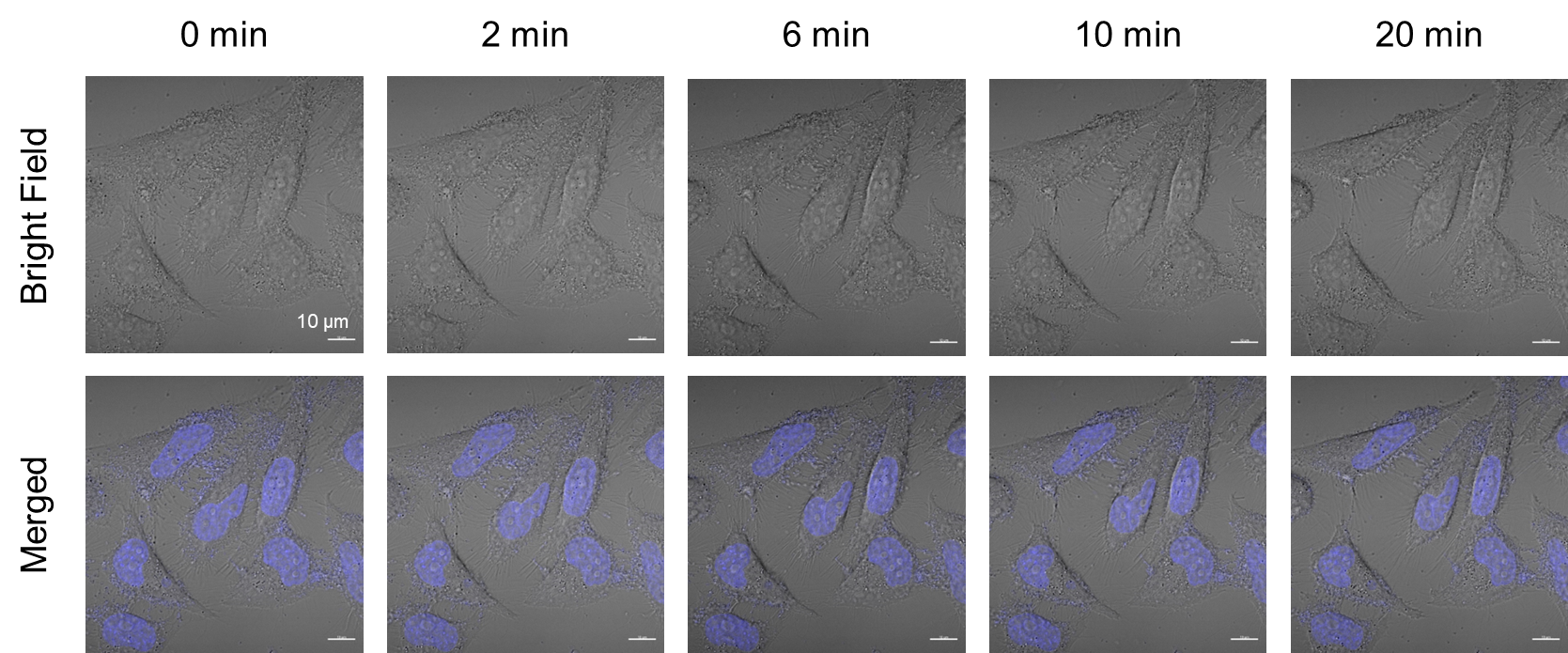


Figure S31. CLSM images of HeLa cells stained with Hoechst under a 405 nm laser (2% power) stimulation with different time.


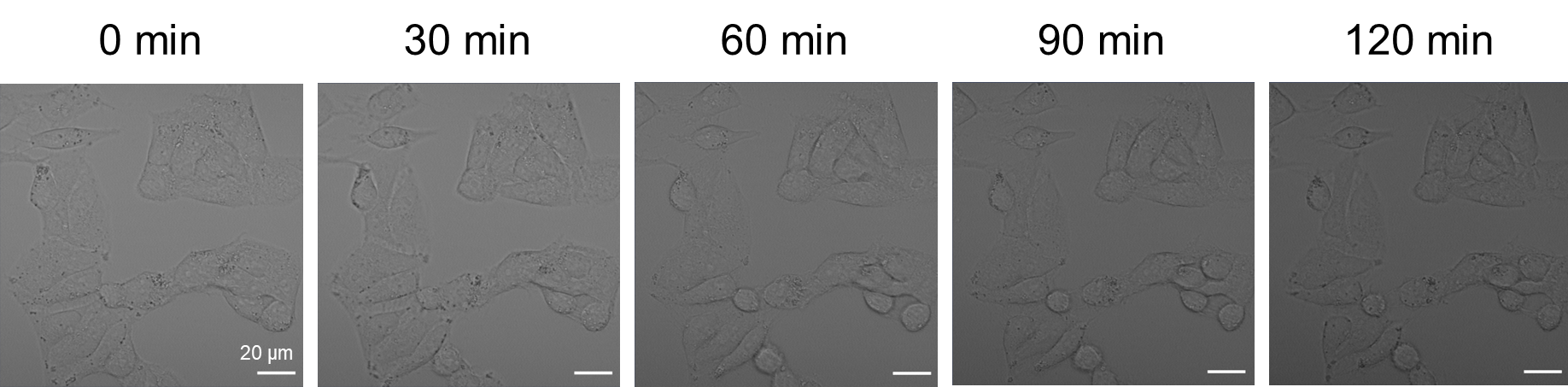


Figure S32. CLSM images of HeLa cells under a 405 nm laser (2% power) stimulation with different time.


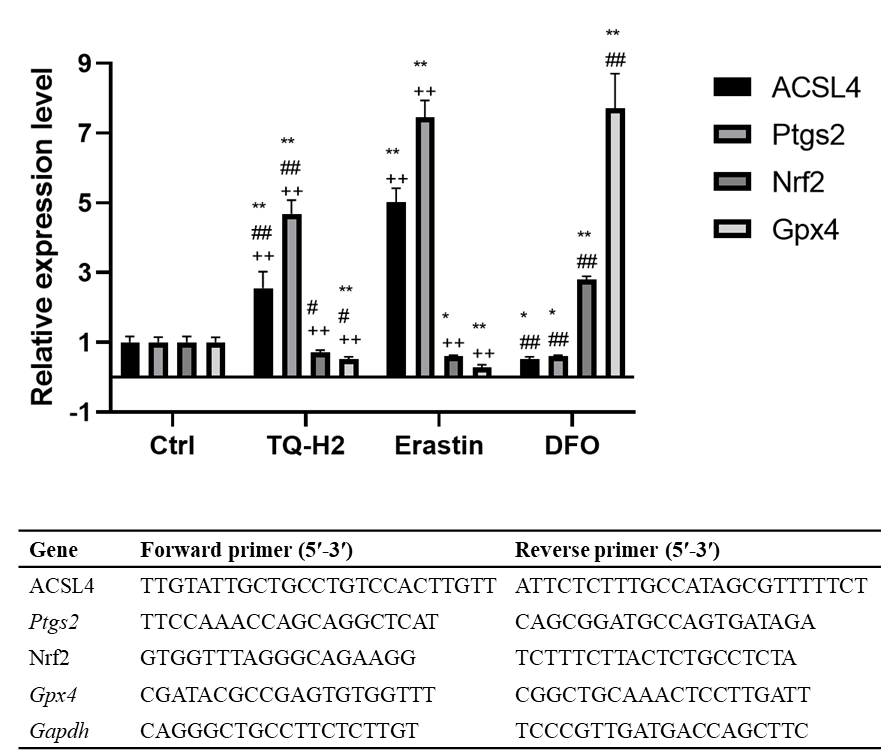


Figure S33. Quantitative real-time PCR analysis of ACSL4, Ptgs2, Nrf2 and Gpx4 mRNA transcription. Using GAPDH as an internal control to standardize the data, the results showed folding change compared with the control group. There were four groups of experiments: the control group (Ctrl), the TQ-H2 group (TQ-H2), the ferroptosis promotion group (Erastin), and the ferroptosis suppression group (DFO). For the promotion group, HeLa cells were first incubated with a medium containing Erastin (10 μM) for 16 h. Then, the medium was removed and washed with PBS for three times. For the suppression group, HeLa cells were first incubated with a medium containing DFO (10 μM) for 18 h. Then, the medium was removed and washed with PBS for three times. Afterwards, the cells were stained with TQ-H2 (10 μM) for 20 mins. Real-time qPCR was performed to detect the levels of ferroptosis-related genes (ACSL4, Ptgs2, Nrf2 and Gpx4). Total RNA was isolated from cells with TRIzol (Invitrogen, USA) and then reverse transcribed into cDNA using a reverse transcription kit (TaKaRa, Japan). The quantity of the mRNA was measured using a Super SYBR qPCR Master Mix kit (ES Science, China) and was performed in an ABI Prism 7500 real-time thermocycler (Applied Biosystems, USA). GADPH was used as an internal reference. Results were calculated using the 2−ΔΔCt method.


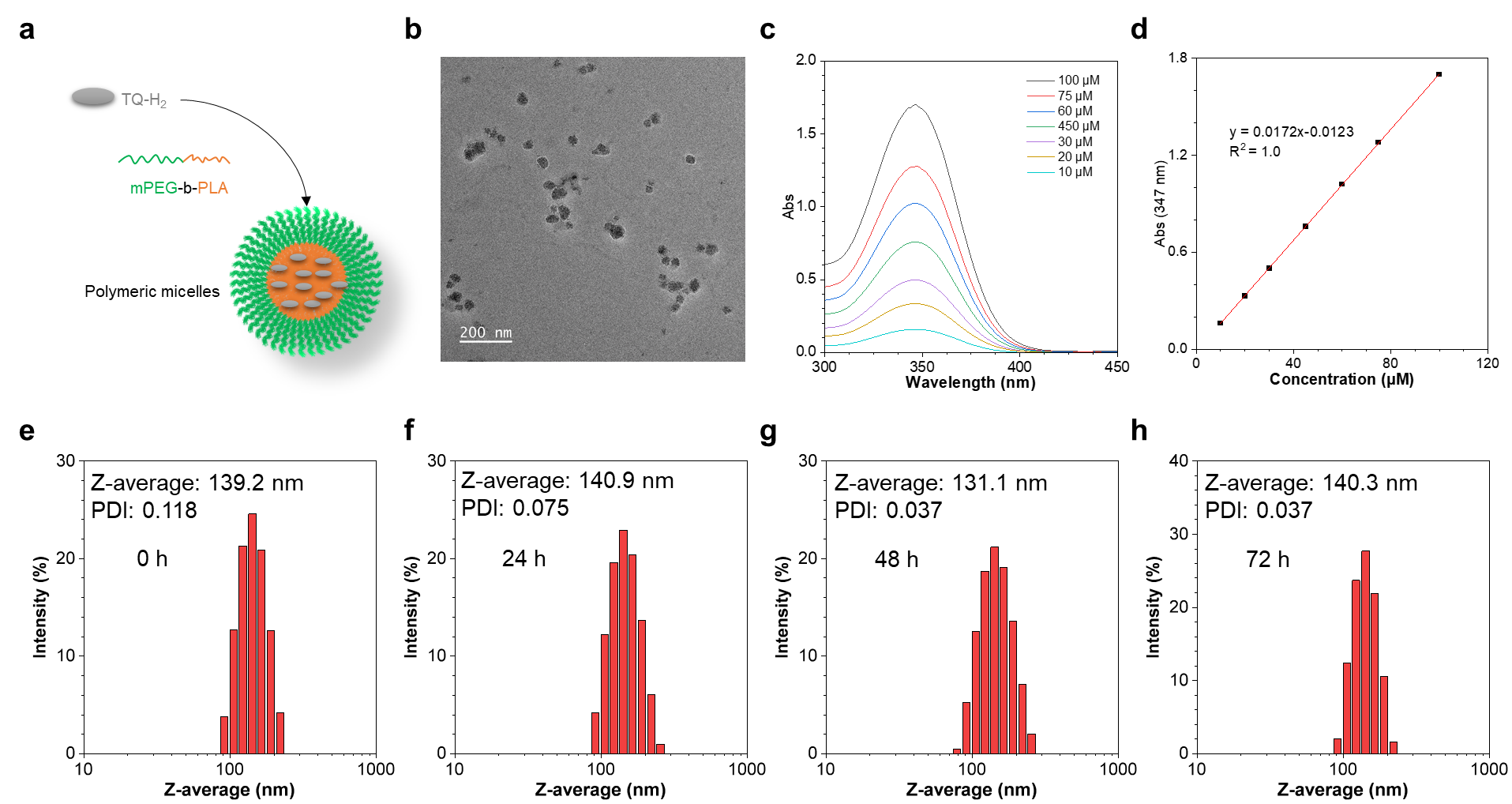


Figure S34. (a) Schematic illustration of the preparation of TQ-H_2_ micelles. (b) TEM of TQ-H_2_ micelles. (c) The absorbance curves of TQ-H_2_ at different concentrations. (d) The standard curve of concentration and absorbance (347 nm) of TQ-H_2_. (e-h) Size distribution of TQ-H_2_ micelles with different time.


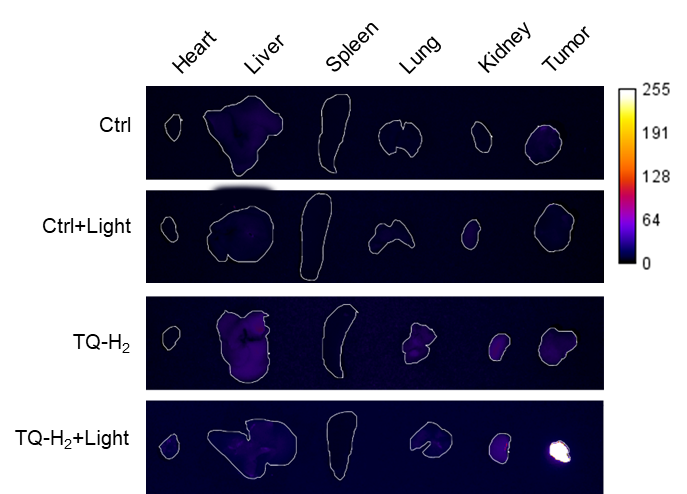


Figure S35. Fluorescence images of organs of mice at different treatment conditions. Excitation wavelength = 505 nm. Emission wavelength = 650 nm.


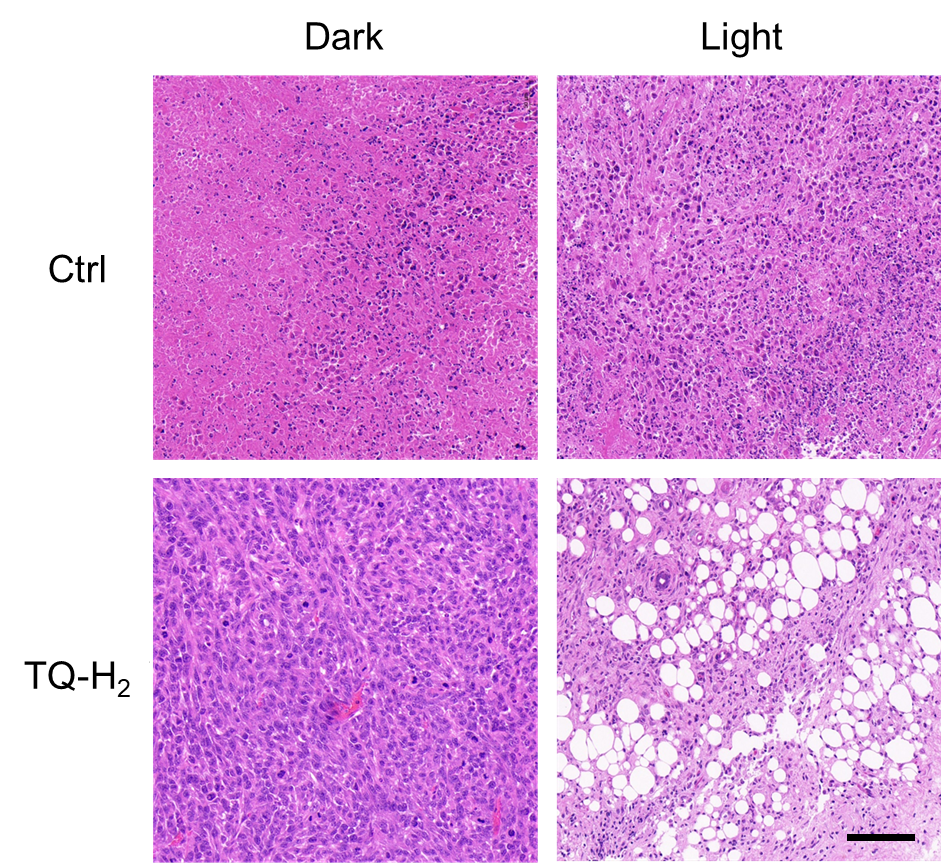


Figure S36. Immunohistochemical analysis of hematoxylin-eosin staining in tumor sections of mice at different treatment conditions. The mice were treated with TQ-H_2_ (10 mg/kg) and physiological saline with or without light irradiation.


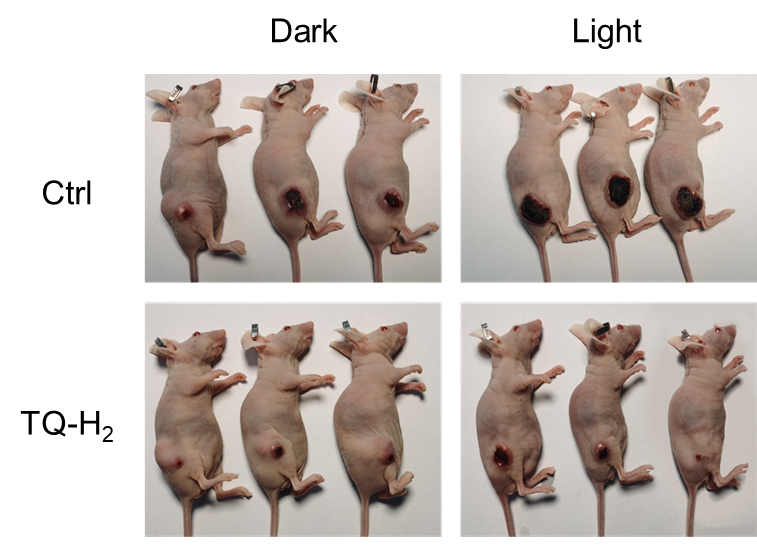


Figure S37. The representative graphs of nude mice in different treatment groups.


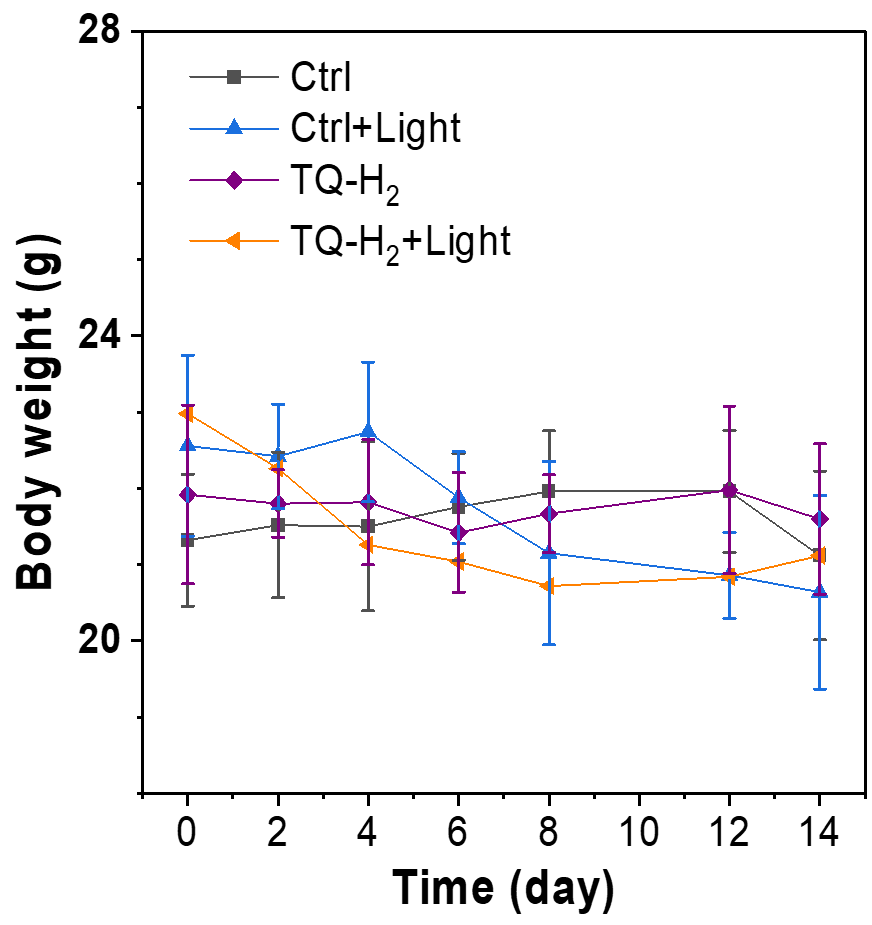


Figure S38. The weight change of mice in different treatment groups during the therapeutic process.


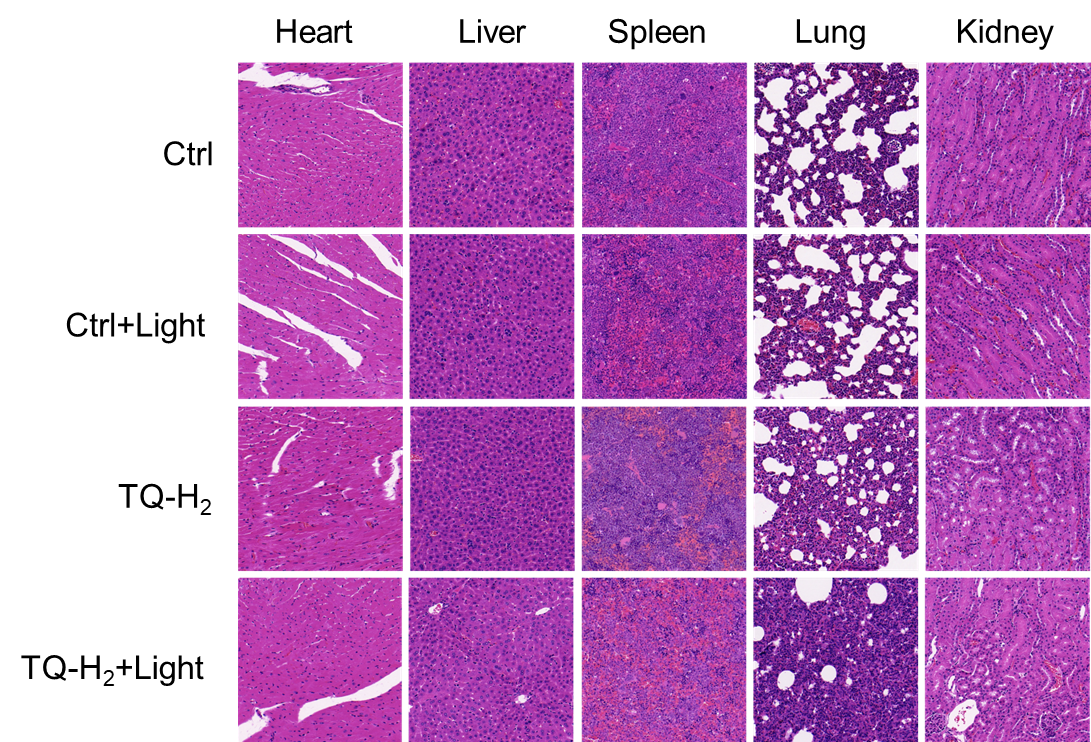


Figure S39. Immunohistochemical analysis of hematoxylin-eosin staining in organs of mice at different treatment conditions.
